# Pan-cancer analysis reveals interleukin-17 family members as biomarkers in the prediction for immune checkpoint inhibitor curative effect

**DOI:** 10.3389/fimmu.2022.900273

**Published:** 2022-09-08

**Authors:** Xiaying Han, Jianxin Ye, Runzhi Huang, Yongai Li, Jianpeng Liu, Tong Meng, Dianwen Song

**Affiliations:** ^1^ Department of Orthopedics, Shanghai General Hospital, Shanghai Jiaotong University School of Medicine, Shanghai, China; ^2^ Department of Surgery, The Second Affiliated Hospital, Zhejiang University School of Medicine, Hangzhou, China; ^3^ Department of Orthopedics, The First Affiliated Hospital of Zhengzhou University, Zhengzhou, China; ^4^ Division of Spine, Department of Orthopedics, Tongji Hospital Affiliated to Tongji University School of Medicine, Shanghai, China; ^5^ Department of Pathology, The First Affiliated Hospital of Wenzhou Medical University, Wenzhou, China

**Keywords:** IL-17 family, pan-cancer, TCGA, systematic analysis, immunotherapeutic effects

## Abstract

**Background:**

The interleukin-17 (IL-17) family contains six homologous genes, IL-17A to IL-17F. Growing evidence indicates that dysregulated IL-17 family members act as major pathogenic factors in the early and late stages of cancer development and progression. However, the prevalence and predictive value of IL-17 for immune checkpoint inhibitor (ICI) therapeutic effectiveness in multiple tumor types remain largely unknown, and the associations between its expression levels and immunotherapy-associated signatures also need to be explored.

**Methods:**

The pan-cancer dataset in The Cancer Genome Atlas (TCGA) was downloaded from UCSC Xena (http://xena.ucsc.edu/). The immunotherapeutic cohorts included IMvigor210, which were obtained from the Gene Expression Omnibus database and included in a previously published study. Other datasets, namely, the GEO dataset and PRECOG, GEO, and METABRIC databases, were also included. In 33 TCGA tumor types, a pan-cancer analysis was carried out including their expression map, clinical risk assessment, and immune subtype analysis, along with their association with the stemness indices, tumor microenvironment (TME) in pan-cancer, immune infiltration analysis, ICI-related immune indicators, and drug sensitivity. RT-PCR was also carried out to verify the gene expression levels among MCF-10A and MCF-7 cell lines.

**Results:**

The expression of the IL-17 family is different between tumor and normal tissue in most cancers, and consistency has been observed between gene activity and gene expression. RT-PCR results show that the expression differences in the IL-17 family of human cell (MCF-10A and MCF-7) are consistent with the bioinformatics differential expression analysis. Moreover, the expression of the IL-17 family can be a sign of patients’ survival prognosis in some tumors and varies in different immune subtypes. Moreover, the expression of the IL-17 family presents a robust correlation with immune cell infiltration, ICI-related immune indicators, and drug sensitivity. High expression of the IL-17 family is significantly related to immune-relevant pathways, and the low expression of IL-17B means a better immunotherapeutic response in BLCA.

**Conclusion:**

Collectively, IL-17 family members may act as biomarkers in predicting the prognosis of the tumor and the therapeutic effects of ICIs, which provides new guidance for cancer treatment.

## Introduction

The interleukin-17 (IL-17) family consists of a subset of cytokines involved in acute and chronic inflammatory responses. These members play crucial roles in host defense against microorganisms and the development of inflammatory diseases, which have gained a lot of attention recently (1, 2). Since the discovery of IL-17A in 1993 (3), other IL-17A homologous genes, namely, IL-17B, IL-17C, IL-17D, IL-17E (also known as IL-25), and IL-17F, have been screened and identified (4). As early as 1863, Virchow considered that chronic inflammation and the incidence of cancer were indeed closely related (5). In detail, chronic inflammation can trigger a series of molecular events, leading to malignant transformation of differentiated cells and antitumor immunosuppression, ultimately leading to tumor occurrence and metastasis (6–9). Thus, IL-17 has been intensively studied in the context of cancer development and progression. Its dysregulation is regarded as a major pathogenic factor involved in the early and late stages of cancer development (2). If targeted respectively, the IL-17/L-17R axis could serve as a potential novel immunotherapeutic target in cancer (10, 11). For example, IL-17 has been reported to be involved in the initiation and development of pancreatic precursor lesions of pancreatic ductal adenocarcinoma (PDAC) (12). The interaction between IL-17 and IL-17RA, which are overexpressed in the epithelium upon Kras activation, promotes a stemness signature in premalignant lesions, and IL-17 blockade increases immune checkpoint inhibitor (ICI) [cytotoxic T lymphocyte antigen 4 (CTLA-4) and programmed death 1 (PD-1)] sensitivity (13, 14). However, the predictive value of the IL-17 family for ICIs’ curative effect in multiple tumor types remains largely unknown, and it is also necessary to explore the relationship between its expression level and immunotherapy-related signatures.

Novel immune checkpoints are expressed on immune cells, can regulate the degree of immune activation, and play an important role in preventing the occurrence of autoimmunity. The abnormal expression and function of immune checkpoints are one of the important reasons for the occurrence of many diseases. ICIs could significantly prolong the overall survival (OS) of cancer patients who were sensitive to ICI therapy (15, 16), and a majority of patients fail to benefit from ICI treatment (17, 18). Therefore, there is a substantial unmet clinical need in identifying suitable patients who may respond to treatment with ICIs (19–21).

In this study, the IL-17 expression landscape of 33 different cancers was presented and the underlying effect of IL-17 expression in tumor prognosis, tumor immune microenvironment, and tumor immunotherapy has been explored. Many important immune modulators and dynamic immunological biomarkers, such as Tumor Immune Dysfunction and Exclusion (TIDE) value, PD-L1 expression, tumor mutational burden (TMB), and microsatellite instability (MSI), were investigated in this research. Furthermore, the correlation between IL-17 expression and ICI treatment was explored. Overall, this work provided evidence to elucidate the immunotherapeutic role of IL-17 in cancer, which could provide new guidance for cancer treatment.

## Methods and materials

### The mRNA expression of IL-17A–F in single cell type and normal human tissues

The Human Protein Atlas (https://www.proteinatlas.org/) was used to explore the expression of IL-17A–F in single cell types and normal human tissues.

### Data download and preprocessing

The pan-cancer dataset in The Cancer Genome Atlas (TCGA) was downloaded from UCSC Xena (http://xena.ucsc.edu/). Firebrowse (http://firebrowse.org/) was used to analyze the basic features of the pan-cancer dataset. For the therapeutic cohort, a systematic search was performed to identify the ICI cohorts, which could be publicly retrieved and reported with complete clinical information. An immunotherapeutic cohorts were finally employed in this study: advanced urothelial cancer with atezolizumab intervention (IMvigor210 cohort downloaded from previously published research) (22). The full names and abbreviations of 33 types of tumors in the GDC TCGA from the UCSC Xena database are shown in **Table S1**.

### Differential expression, coexpression, and gene activity of IL-17A–F in tumor and normal tissues

For all types of TCGA tumors, “ggpubr” R package was used for differential expression analysis between tumor and adjacent tissues (Wilcox test). The difference in gene expression of the IL-17 family in pan-cancer showed log2 fold change (log2FC) in heatmap. The Corrplot R software package was used to analyze the coexpression of IL-17A–F, and the protein–protein interaction (PPI) network between these genes and their receptor genes was also constructed by applying STRING database (https://string-db.org/) (23) and Cytoscape (Version: 3.8.2). IL-17B, IL-17C, and IL-17D gene activity was generated by single sample gene set enrichment analysis (ssGSEA). First, the average values of the gene activity of 33 cancers were calculated and arranged. After that, the differences in gene activity in tumors and normal tissue were explored.

### Clinical correlation analysis

The differences in OS results between patients with high and low expression of IL-17A–F were analyzed; Kaplan–Meier plots of IL-17 genes in pan-cancer were generated using the R package (24, 25). The phenotype information and survival outcome data of 33 TCGA cancer types were downloaded from the GDC TCGA dataset in the UCSC Xena database. According to the median expression levels of IL-17A–F, patients were divided into a high-expression group and a low-expression group. In addition, we also performed Cox proportional hazard regression to analyze the hazard ratios of IL-17A–F in each TCGA tumor type. Moreover, the differences in IL-17 expression levels in different stages of KIRP and BRCA were analyzed. Variations with a *p*-value < 0.05 were considered significant.

### Immune subtype analysis

Tumor microenvironment (TME) had an important therapeutic and prognostic significance in antitumor therapy. Based on five representative immune signals, researchers identified six immune subtypes of TCGA tumors, which provided resources for the analysis of TME in some specific tumors. Six stable and repeatable immune subtypes were classified according to five immune expression characteristics (26): macrophages/monocytes (27), total lymphocyte infiltration (mainly T and B cells) (28), TGF-β response (29), IFN-γ response (30), and wound healing (31). The six subtypes may play a key role in predicting disease outcomes, rather than relying solely on the characteristics of individual cancer types (26). In order to detect the mRNA expression levels of IL-17A–F of six different immune subtypes in TCGA tumor types, we used Kruskal–Wallis test for differential expression analysis.

### Stemness indices and TME in pan-cancer

Solid tumor tissue is composed of tumor cells and non-tumor cells such as immune cells, stromal cells, and vascular cells. To study the association between the expression level of IL-17 and the proportion of immune and stromal cells in TCGA tumor types, ESTIMATE (estimation of immune scores and stromal scores) (32) was utilized to reckon the proportion of these two TME components. The ESTIMATE scores were calculated according to the characteristics of gene expression, which reflected the purity of the tumor to a certain extent. Therefore, the Spearman correlation between the expression level of IL-17 genes and stromal scores was analyzed by using the ESTIMATE package and the limma package.

In order to further analyze the correlation in pan-cancer between the IL-17 family and stemness characteristics, one-class logistic regression (OCLR) algorithm was used and Spearman correlation analysis was performed based on gene expression and stemness indices (33). Stemness indices described the potential renewal ability of cells, which could lead to tumor metastasis and occurrence. Here, we used the DNA methylation-based stemness index (DNAss) and the mRNA expression-based stemness index (RNAss), which were two types of stemness indices.

For STAD and THCA, specifically, we utilized RNAss, DNAss, stromal scores, immune scores, and ESTIMATE scores to analyze the correlation with IL-17 transcriptional expression.

### Immune infiltration analysis

The composition and abundance of immune cells in the TME strongly influence the progress of tumors and the effect of immunotherapy (34). TIMER2.0 no longer uses only one algorithm (http://timer.cistrome.org/) but uses the six most advanced algorithms, namely, TIMER, CIBERSORT, quanTIseq, xCell, MCP-counter, and EPIC algorithms, to provide more reliable immune infiltration levels for TCGA or tumor profile by using the estimated infiltration level (35, 36). The correlation of the expression of IL-17A/B/F genes and the level of immune infiltration of different tumor types was explored. The scores of six immune infiltrating cells of 33 tumors were obtained in the TIMER2.0 database, the correlation between IL-17A/B/F gene expression and these immune infiltrating cells was analyzed, and the correlation between IL-17B transcription level and the purity of BLCA, CESC, KIRC, and PCPG was also obtained at the same time.

### Cytotoxic T-lymphocyte infiltration level and survival analysis

Predicting tumor response to immune checkpoint blockers (ICBs) requires an understanding of how tumors escape the immune system. We used the web application TIDE (http://tide.dfci.harvard.edu) to analyze the related work (37). The cancer datasets with both patient survival durations and tumor gene expression profiles from the TCGA (38), PRECOG (39), GEO, (40) and METABRIC (41) databases (37). The PRECOG database provided only survival duration information without other clinical factors. METABRIC is a breast cancer cohort, and all tumors were split according to the PAM50 subtypes (luminal A, luminal B, HER2, basal, and triple-negative) (37). The GEO dataset included GSE1427, GSE12417_GPL96, GSE9893, GSE5123, GSE5828, GSE50081, GSE31245, GSE1993, GSE71014, GSE11969, GSE39582, GSE4475, GSE37745, GSE3149, GSE16131, GSE54236, GSE50081, GSE49997, GSE63885, GSE1037, GSE32062, GSE71187, GSE65858, GSE54236, GSE10245, GSE42127, and GSE18521.

To predict each tumor’s potential to escape T cell-mediated killing, we first classified each tumor into CTL (cytotoxic T lymphocytes)-high or CTL-low categories through the CTL marker expression levels (CD8A, CD8B, GZMA, GZMB, and PRF1). The correlation between the expression of IL-17A–F and the CTL infiltration level was explored in different tumors. The association between the CTL level and OS was computed through the two-sided Wald test in the Cox-PH regression. Samples were classified into two groups for each Kaplan–Meier plot: “High CTL” samples have above-average CTL values among all samples, while “Low CTL” samples are below average. Samples were split into a high-expression group and a low-expression group according to the IL-17A–F expression to show the association between the CTL level and survival outcome.

### T-cell dysfunction and exclusion analysis

Here, both T-cell dysfunction and exclusion signatures were used to model immune escape in tumors with different CTL levels (42, 43). Some tumors have a high level of infiltration by cytotoxic T cells, but these T cells tend to be in a dysfunctional state. Patients with dysfunctional T-cell infiltration are more likely to be resistant to ICB reprogramming. In other tumors, immunosuppressive factors may exclude T cells from infiltrating tumors such as impaired priming of tumor-specific T cells or suppressive cells prohibiting T-cell infiltration into the tumor (42–44). The average expression profiles of cancer-associated fibroblasts (CAFs), myeloid-derived suppressor cells (MDSCs), and the M2 subtype of tumor-associated macrophages (TAMs) reported to restrict T-cell infiltration in tumors were to model T-cell exclusion (43). In Jiang’s study, it was described that “the Pearson correlation with either T cell dysfunction (in CTL-high tumors) or exclusion (in CTL-low tumors) signatures was defined as the TIDE prediction score (37)“. All tumors were ranked by their TIDE scores to predict their ICB response. A higher tumor TIDE prediction score is associated not only with worse ICB response, but also with worse patient survival under anti-PD1 and anti-CTLA4 therapies (37). TIDE (http://tide.dfci.harvard.edu) was used to get the related results. The dataset from the TCGA database, E-MTAB-179, GSE12417_GPL570 from the GEO E-MTAB-179 dataset, METABRIC database, Braun2020_PD1 (45), Gide2019_PD1 (46), Hugo2016_PD1 (47), Lauss2017_PD1 (48), Liu2019_PD1 (49), Mariathasan2018_PDL1 (50), Miao2018_ICB (51), Nathanson2017_CTLA4 (52), Riaz2017_PD1 (53), VanAllen2015_CTLA4 (54), and Zhao2019_PD1 (55) were included. The correlation between IL-17A–F gene expression and T dysfunction value in the core dataset, the normalized *z* score calling from Cox-PH regression in the immunotherapy dataset, and the normalized expression value from immune-suppressive cell types were explored.

### Regulation of immune molecule analysis

The TISIDB database (http://cis.hku.hk/TISIDB) integrates 988 reported genes related to antitumor immunity through the analysis of high-throughput screening and genomic analysis data to pre-calculate the association between TCGA cancer-type genes and immune characteristics, including lymphocytes, immunomodulators, and chemokines (56). In our study, we used the TISIDB database to analyze the association between the expression level of IL-17D and immunomodulators. We used the TCGA WES data to calculate the TMB levels in various tumors through the R software package “maftools”, and the MSI scores for each TCGA cancer case were derived from (57), using radar charts to illustrate the relationship between them. Finally, for STAD, we performed gene set enrichment analysis (GSEA) to describe the significant differences observed between the high-expression IL-17 group and the low-expression IL-17 group in KEGG and GO databases. The selected pathway criteria are *p* < 0.05 and the top five highest normalized enrichment scores.

### Immunotherapeutic response analysis

Complete remission (CR), partial remission (PR), progressive disease (PD), and stable disease (SD) are the four outcomes of immunotherapy. In this study, CR and PR were classified as responders, and PD and SD were classified as non-responders. CR, PR, and SD were classified as clinically harmless groups, and PD was assigned to another group. The Wilcoxon test was used to study differences in gene expression of IL-17 members between responders and non-responders in the related independent immunotherapy cohorts (IMvigor 210).

### Drug sensitivity analysis in pan-cancer

The IC_50_ of 265 small molecules in 860 cell lines and its corresponding mRNA gene expression were collected from Genomics of Drug Sensitivity in Cancer (GDSC) (https://www.cancerrxgene.org/) (58–60). The mRNA expression data and drug sensitivity data were merged. Pearson correlation analysis was performed to obtain the correlation between gene mRNA expression and drug IC_50_. *p*-value was adjusted by FDR. The IC_50_ of 481 small molecules in 1,001 cell lines and its corresponding mRNA gene expression were also collected from the Cancer Therapeutics Response Portal (CTRP) (https://portals.broadinstitute.org/ctrp/). The mRNA expression data and drug sensitivity data were merged. Pearson correlation analysis was performed to obtain the correlation between gene mRNA expression and drug IC_50_. *p*-value was adjusted by FDR. A bubble plot was provided to summarize the correlations between inputted genes and drugs. A gene will be obtained only when it is associated with at least one drug. Also, a drug will be obtained only when it is associated with at least one gene.

### Quantitative real-time polymerase chain reaction assay

Total RNA was extracted from MCF-10A and MCF-7 cell lines with the TRIzol kit (Invitrogen, Carlsbad, CA, USA), the obtained RNA was reverse transcribed into cDNA with a reverse transcription kit (EnzyArtisan, Shanghai, China), and a SYBR Green qPCR Kit (EnzyArtisan, Shanghai, China) was picked to amplify the target transcript to analyze gene expression. The human primer sequences for IL-17B, IL-17C, IL-17D, and IL-25 (IL-17E) are listed in **Table S2**.

## Results

### The mRNA expression of IL-17A–F in single-cell type and normal human tissues

**Figures S1**, **S2** show the mRNA expression of IL-17A–F in single-cell type and normal human tissues. IL-17A is mainly expressed in T cells including naive CD4 T-cell activated, memory CD4 T-cell Th17, naive CD8 T-cell activated, memory T-reg, memory CD4 T-cell TFH, dendritic cells, Langerhans cells, macrophages, and gastric mucus-secreting cells. Among all organs, it is mainly expressed in bone marrow and lymphoid tissues, kidney and urinary bladder, liver and gallbladder, the gastrointestinal tract, and the proximal digestive tract. IL-17B is mainly expressed in breast myoepithelial cells, Sertoli cells, peritubular cells, theca cells, smooth muscle cells, late spermatids, cardiomyocytes, fibroblasts, early spermatids, Langerhans cells, Kupffer cells, breast glandular cells, basal keratinocytes, and extravillous trophoblasts. Among all organs, it is mainly expressed in male/female tissues, muscle tissues, the proximal digestive tract, the gastrointestinal tract, endocrine tissues, bone marrow, and lymphoid tissues. IL-17C is mainly expressed in basal respiratory cells, club cells, ionocytes, respiratory ciliated cells, cholangiocytes, and exocrine glandular cells. Among all organs, it is mainly expressed in male/female tissues, kidney and urinary bladder, liver, and gallbladder. IL-17D is mainly expressed in astrocytes, late spermatids, oligodendrocyte precursor cells, Muller glia cells, early spermatids, excitatory neurons, oligodendrocytes, inhibitory neurons, Leydig cells, skeletal myocytes, melanocytes, spermatocytes, fibroblasts, extravillous trophoblasts, rod photoreceptor cells, and microglial cells. Among all organs, it is mainly expressed in brain and skeletal muscle. IL-25 is mainly expressed in cardiomyocytes. Among all organs, it is mainly expressed in testis and muscle tissues. IL-17F is mainly expressed in T cells (naive CD8 T cells). Among all organs, it is mainly expressed in bone marrow and lymphoid tissues, kidney, and urinary bladder.

### Differential expression, coexpression, and gene activity of IL-17A–F in tumor and normal tissues

**Figure 1:**
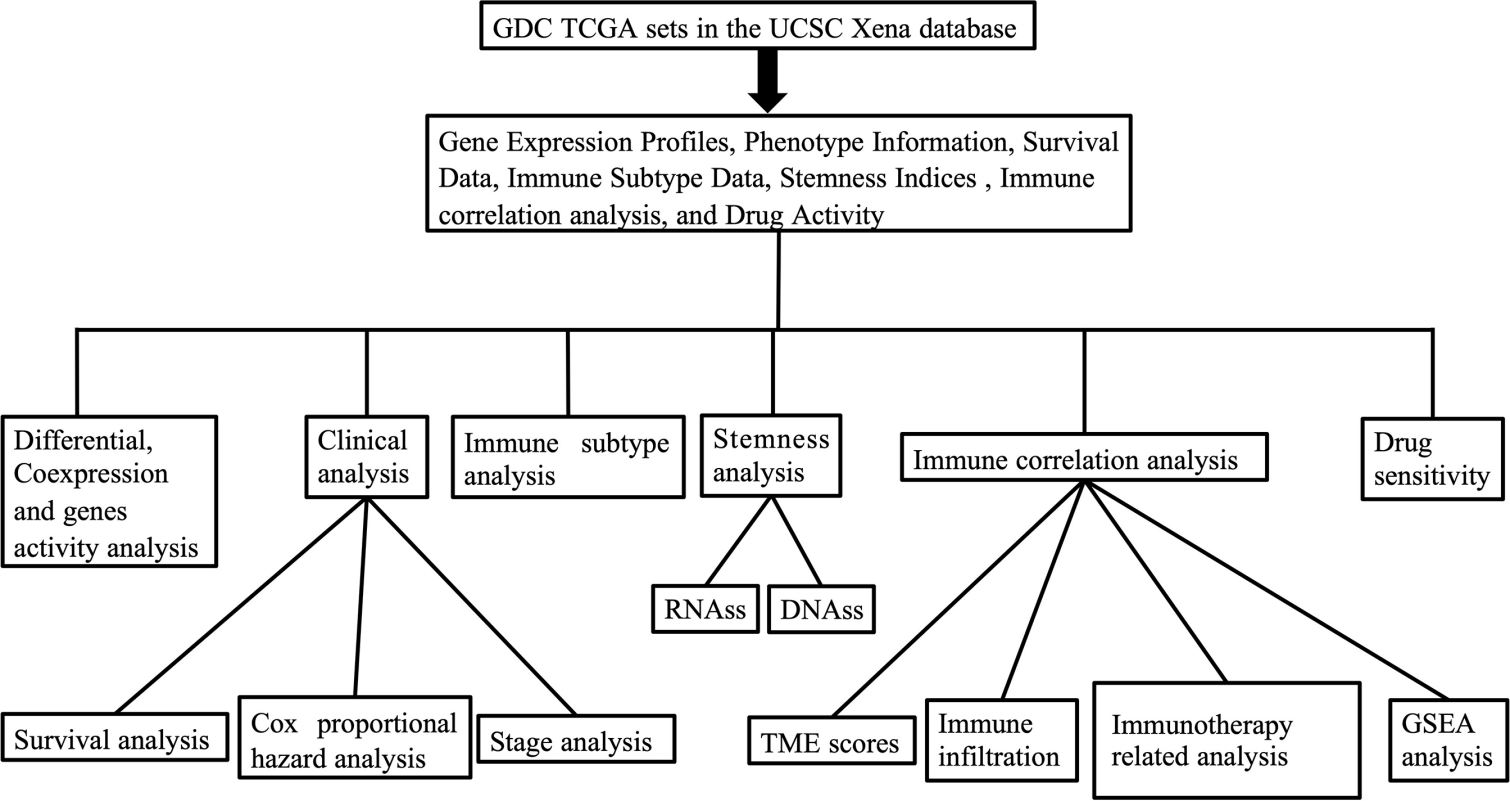
The outline of our research.

**Figure 2:**
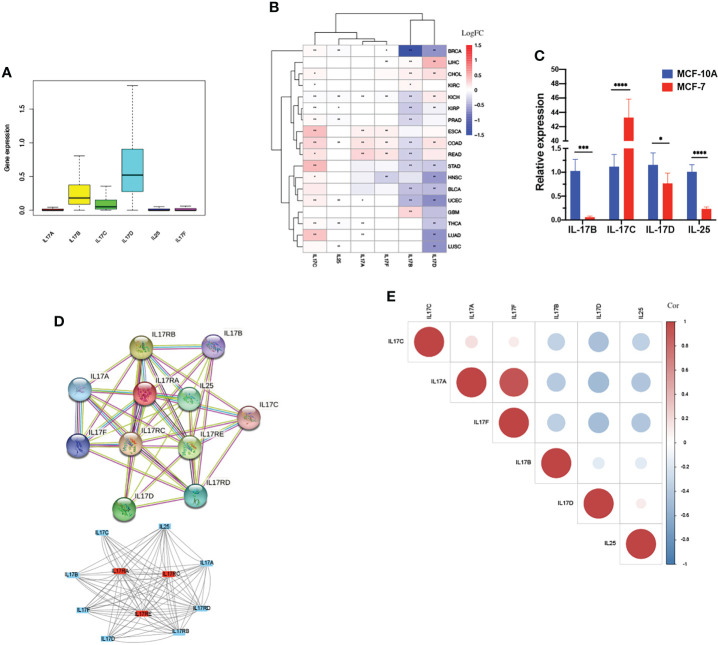
Differential expression analysis. **(A)** The box plot showing the transcriptional expression levels of the IL-17 family based on the TCGA dataset. **(B)**The heatmap exhibiting the transcriptional level of the IL-17 family in TCGA tumor types compared to adjacent normal tissues; the gradient colors represent the log fold change (logFC) value. (Red points indicate high expression, while blue points indicate low expression.) **(C)** Level of IL-17B, IL-17C, IL-17D, and IL-25 (IL-17E) mRNA in MCF-10A and MCF-7. **(D)** The protein–protein interaction (PPI) network constructed among IL-17A, IL-17B, IL-17C, IL-17D, IL-25, IL-17F, and their receptors. The core gene was marked red. **(E)** Co-expression analysis between each two genes is presented. (Red points indicate positive correlation, while blue points indicate negative correlation.) (∗∗∗∗*p* < 0.0001; ∗∗∗*p* < 0.001; ∗∗*p* < 0.01; ∗*p* < 0.05.).

**Figure 3:**
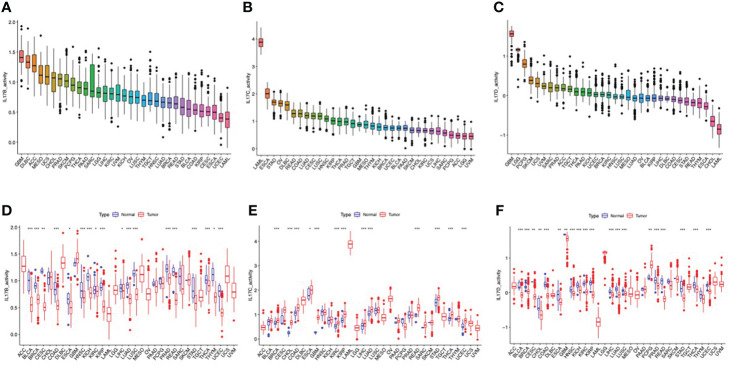
The different activity analyses based on the TCGA dataset. **(A–C)** The mean activity of IL-17B, IL-17C, and IL-17D in 33 cancers (from high to low). **(D–F)** The different activity analysis between tumors and normal groups of IL-17B, IL-17C, and IL-17D in 33 cancers (from high to low). (∗∗∗∗*p* < 0.0001; ∗∗∗*p* < 0.001; ∗∗*p* < 0:01; ∗*p* < 0.05.).

The outline of our research is shown in **Figure 1**. We used datasets from TCGA, and the basic information is shown in **Figure S3**. The gene expression of IL-17A–F is displayed in **Figure 2A**. Wilcox test was used to analyze the differential expression of five IL-17 family genes in tumor and adjacent tissue (**Figure 2B**). Obviously, the expression of IL-17D was the highest among the IL-17 family in major tumors. Across the heatmap, we could find that the expression of IL-17C was mostly upregulated in most types of tumors, while the expression of IL-17B and IL-17D was low in some tumors. Compared to normal tissues, the expression of IL-17B was downregulated in most tumors except CHOL, GBM, and LIHC (*p* < 0.01), and the expression of IL-17C was upregulated in all tumors except KICH (*p* < 0.01). Moreover, IL-17D was the only gene in the IL-17 family whose expression was downregulated in LUAD (*p* < 0.01). In the three gastrointestinal cancers including ESCA, COAD, and READ, the expression of IL-17F was all upregulated (*p* < 0.01). IL-17B was significantly downregulated in BRCA compared to normal tissues. Furthermore, we once again performed *in vitro* experiments to investigate the expression differences of IL-17B, IL-17C, IL-17D, and IL-25 (IL-17E) of human cell (MCF-10A and MCF-7) (**Figure 2C**), which was consistent with the differential expression analysis mentioned above. Coexpression analysis showed a strongly positive coexpression relationship (*r* = 0.71) between IL-17A and IL-17F (**Figure 2E**), further proved by the PPI network (**Figure 2D**). The contact could also be revealed between IL-17A and IL-17C (*r* = 0.2). On the contrary, there was a negative coexpression relationship between IL-17D and IL-17C (*r* = −0.1). As regards gene activity, the difference in gene activity between normal tissues and adjacent tissues was basically consistent with the difference in gene expression (**Figure 3**). For example, the gene activity of IL-17B in BRCA was also lower compared with normal tissues.

### Clinical correlation analysis

We performed Kaplan–Meier analysis of IL-17A–F in 33 types of TCGA tumors (**Figures S4**, **S5**). According to the median gene expression levels, the patients were divided into a high-expression group and a low-expression group. In general, as far as the IL-17A gene was concerned, the OS rate of patients with COAD (*p* = 0.009, **Figure S4A**) and HNSC (*p* = 0.010) in the high-expression group was higher than that in the low-expression group, and patients with DLBC (*p* = 0.049) in the low-expression group had a higher survival rate. As far as the IL-17B gene was concerned, the OS rate of patients with KIRP (*p* < 0.001, **Figure S4B**), KIRC (*p* = 0.041), and LGG (*p* = 0.004) in the high-expression group was lower than that in the low-expression group, while the OS rate of patients with BRCA (*p* = 0.007) in the high-expression group was higher than that in the low-expression group. For IL-17C, patients with high IL-17C expression in KIRC (*p* = 0.004, **Figure S4C**) and ACC (*p* = 0.014) have a lower survival rate than patients with low IL-17C expression. For IL-17D, patients with high IL-17D expression in LGG (*p* < 0.001, **Figure S4D**), PAAD (*p* = 0.004), LAML (*p* = 0.004), and LUAD (*p* = 0.025) have a higher survival rate than patients with low IL-17D expression; patients with high IL-17D expression in KICH (*p* = 0.02) and KIRP (*p* = 0.005) have a higher survival rate than patients with low IL-17C expression. The low expression of IL-25 in patients with CESC (*p* = 0.013, **Figure S4E**) and the low expression of IL-17F in patients with KIRC (*p* = 0.01, **Figure S4F**), LUAD (*p* = 0.012), and HNSC (*p* = 0.019) were significantly correlated with worse survival rate. The remaining results are shown in **Supplementary Figure S5**.

Cox proportional hazard regression was also applied to investigate the prognostic function of the IL-17 family in pan-cancer. In the forest plot (**Figure S4G**), we found that IL-17B and IL-17C had important prognostic significance with hazard ratio (HR) >1 in the majority of cancers, which was recognized as a high-risk prognostic factor.

Notably, the expression of IL-17B (*p* < 0.001) and IL-17D (*p* < 0.001) in KIRP was associated with TNM stages. The same monotonous increasing trend was observed in the expression of both IL-17B and IL-17D from stage I to stage IV (**Figure S6A**). The difference was that IL-17B (*p* = 0.004) gene expression was not monotonous in different stages of BRCA, but the highest in stage I, followed by stage III, relatively low in stages II and IV, and the lowest in stage IV (**Figure S6B**). The differential expression of IL-17 family genes in four TNM stages might be used as a predictor of tumor progression.

### Immune subtype analysis

We used Kruskal–Wallis test to analyze the mRNA expression of IL-17A–F genes in six immune subtypes of 33 types of TCGA tumors. The expression levels in C1–C6 of IL-I7A (*p* < 0.001), IL-17B (*p* < 0.001), IL-17C (*p* < 0.001), IL-17D (*p* < 0.001), IL-25 (*p* < 0.001), and IL-17F (*p* < 0.001) have a significant statistical difference (**Figure S7A**). Obviously, IL-17D ranked the first among the five groups. Moreover, the gene expression of different types of TCGA tumors showed differences in immune subtypes. For HNSC (**Figure S7B**), IL-17D (*p* < 0.001) and IL-25 (*p* < 0.01) in C1–C6 showed a similar pattern, with a high expression in C3 and C6, and a relatively low expression in C1, C2, and C4. IL-17B (*p* < 0.05), however, exhibited the highest expression in C4 and the lowest expression in C3. For BRCA (**Figure S7C**) and LUSC (**Figure S7D**), it is undeniable that there was a significant difference in the five members of the IL-17 family in BRCA. The expression of IL-17B (*p* < 0.001) and IL-17D (*p* < 0.001) in BRCA and the expression of IL-17B (*p* < 0.01), IL-17C (*p* < 0.05), and IL-17D (*p* < 0.001) in LUSC were similar to the expression trend of IL-17D in HNSC. In particular, as we can see, the expression of many genes in the immune subtypes of these three tumors was very low; there were many 0 values in the original data.

### Stemness indices and TME in pan-cancer

In order to further analyze the association between the gene expression of the IL-17 family and stemness features of pan-cancer, we used the OCLR algorithm to calculate the stemness indices of TCGA tumor samples (including DNAss and RNAss), and according to IL-17 gene expression and stemness indices, Spearman correlation analysis was performed (33).

**Figure 4:**
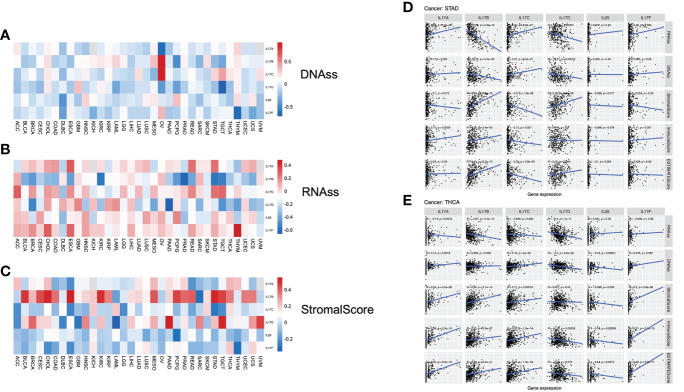
**(A–C)** Correlation analysis between IL-17A–F expression and stemness indices and tumor microenvironment (TME). The association between IL-17A–F expression and DNAss, RNAss, and stromal score in 33 TCGA cancer types. (Red points indicate positive correlation, while blue points indicate negative correlation.) **(D, E)** The association between IL-17A–F expression and stemness indices (RNAss and DNAss), stromal scores, immune scores, and ESTIMATE scores in STAD and THCA in TCGA cancer.

In TCGA tumors, the correlation between the two stemness indices and IL-17 expression levels was different. For DNAss, it is not hard to find that there were obviously positive correlations between OV and IL-17B (*r* = 0.93, *p* = 0.007) and IL-17C (*r* = 0.89, *p* = 0.012). Strongly negative correlation was detected between TGCT and IL-17D (*r* = −0.71, *p* < 0.001) and between THYM and IL-17F (*r* = −0.63, *p* < 0.001) (**Figure 4A**). For RNAss, significantly negative correlations were observed between PAAD and IL-17D (*r* = −0.66, *p* < 0.001) and between PRAD and IL-17B (*r* = −0.61, *p* < 0.001). A positive correlation between THYM and IL-17F (*r* = 0.48, *p* < 0.001) could be seen (**Figure 4B**).

The stromal scores of TCGA cancer samples were calculated by the ESTIMATE (Estimation of STromal and Immune cells in MAlignant Tumors using Expression data) algorithm (32). Therefore, the Spearman correlation between the transcriptional expression of the five IL-17 genes and stromal scores was analyzed. The expression of IL-17B was positively associated with a few types of tumors, containing BLCA, CESC, CHOL, ESCA, KIRC, MESO, PCPG, STAD, and TGCT, suggesting that the high expression of IL-17B may lead to low purity in many types of tumors. The role of IL-17D towards tumor purity was the same as IL-17B, embodied in BRCA, HNSC, PAAD, SARC, TGCT, and UVM (**Figure 4C**).

For STAD (**Figure 4D**), the IL-17A expression was positively correlated with RNAss (*r* = 0.25, *p* < 0.001) and immune scores (*r* = 0.15, *p* = 0.0058). The expression profiles of IL-17B were positively correlated with stromal scores (*r* = 0.53, *p* < 0.001), immune scores (*r* = 0.23, *p* < 0.001), and ESTIMATE scores (*r* = 0.41, *p* < 0.001), while negatively correlated with stemness indices (RNAss: *r* = −0.56, *p* < 0.001; DNAss: *r* = −0.23, *p* < 0.001), and the correlation between IL-17D and these five indices was similar. Interestingly, we found that IL-17C is the opposite of IL-17B; the expression profiles of IL-17C were negatively correlated with stromal scores (*r* = −0.3, *p* < 0.001), immune scores (*r* = −0.12, *p* = 0.027), and ESTIMATE scores (*r* = −0.23, *p* < 0.001), while positively correlated with stemness indices (RNAss: *r* = 0.27, *p* < 0.001; DNAss: *r* = 0.27, *p* < 0.001). In addition, there was a slight and statistically insignificant correlation with stemness indices and tumor purity in both IL-25 and IL-17F.

In THCA (**Figure 4E**), IL-17A, IL-17B, and IL-17C had obvious similarities with the correlation between stemness indices and TME. Taking IL-17A as an example, it is positively correlated with DNAss (*r* = 0.14, *p* = 0.0018), stromal scores (*r* = 0.24, *p* < 0.001), immune scores (*r* = 0.38, *p* < 0.001), and ESTIMATE scores (*r* = 0.35, *p* < 0.001), but negatively correlated with RNAss (*r* = −0.14, *p* = 0.0012). Notably, we found a negative correlation between IL-17D expression and stemness indices (RNAss: *r* = −0.3, *p* < 0.001; DNAss: *r* = −0.2, *p* < 0.001) and immune scores (*r* = −0.16, *p* < 0.001). Significant negative correlation was also exhibited in IL-25. Nevertheless, there was a positive correlation between IL-17F and TME (Stromal: *r* = 0.2, *p* < 0.001; Immune: *r* = 0.31, *p* < 0.001; ESTIMATE: *r* = 0.29, *p* < 0.001).

### Immune infiltration analysis

**Figure 5:**
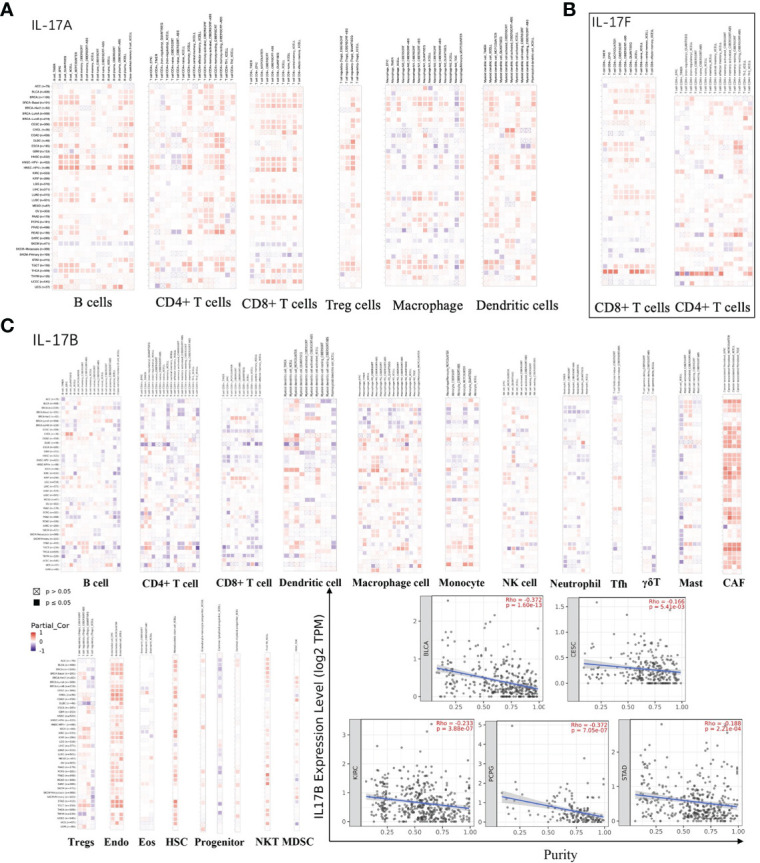
**(A)** The association between IL-17A expression and immune infiltration. **(B)** The association between IL-17F expression and immune infiltration. **(C)** The association between IL-17B expression and immune infiltration, and the purity of BLCA, CESC, KIRC, PCPG, and STAD in TCGA cancer.

The cellular components of TME were considered to regulate the characteristics of cancer and may be used as targets for tumor therapy (61, 62). The expression of IL-17A and IL-17F was positively correlated with immune cells, which secreted them in most tumors (**Figures 5A, B**). The expression of IL-17B was positively correlated with the infiltration level of various immune infiltrating cells including CAF, Endo, HSC, and NKT (**Figure 5C**). However, such an obvious positive correlation was not seen in B cells, CD4^+^ T cells, CD8^+^ T cells, and dendritic cells, especially in DLBC, HNSC, TGCT, and THYM; the trend of this correlation was slightly different, which may be caused by different immune cell infiltration in different cancers. Moreover, we found that the expression of IL-17B was positively correlated with BLCA (*r* = −0.372, *p* < 0.001), CESC (*r* = −0.166, *p* = 0.0054), KIRC (Cor = −0.233, *p* < 0.001), PCPG (*r* = −0.372, *p* < 0.001), and STAD (Cor = −0.188, *p* < 0.001) in stromal scores (**Figure 4C**), indicating that it may be negatively related to tumor purity, which was just confirmed again in **Figure 5C**.

### Cytotoxic T-lymphocyte infiltration and survival level analysis

The results of correlation analysis between CTL level and expression of IL-17A–F are shown in **Figure S8**. Only significant correlations are shown. For BRCA, the expression of IL-17A was positively correlated with the CTL level (GSE9893: *r* = 0.8, *p* < 0.05), and the expression of IL-17F was positively correlated with the CTL level (GSE9893: *r* = 0.64, *p* < 0.05). For GBM, the expression of IL-17B was positively correlated with the CTL level (GSE1993: *r* = 0.304, *p* < 0.05). For LAML, the expression of IL-17A was positively correlated with the CTL level (GSE1427: *r* = 0.483, *p* < 0.05; GSE12417_GPL96: *r* = 0.325, *p* < 0.05), the expression of IL-17B was positively correlated with the CTL level (GSE1427: *r* = 0.324, *p* < 0.05), the expression of IL-17C was positively correlated with the CTL level (GSE71014: *r* = 0.334, *p* < 0.05), and the expression of IL-25 was positively correlated with the CTL level (GSE1427: *r* = 0.401, *p* < 0.05). For LUAD, the expression of IL-17C was positively correlated with the CTL level (GSE11969: *r* = 0.46, *p* < 0.05). For melanoma, the expression of IL-17C was positively correlated with the CTL level (GSE22153: *r* = 0.357, *p* < 0.05). For NSCLC, the expression of IL-17A was positively correlated with the CTL level (TCGA: *r* = 0.355, *p* < 0.05; GSE50081: *r* = 0.387, *p* < 0.05) and negatively correlated with the CTL level (GSE5123: *r* = −0.378, *p* < 0.05; GSE5123: *r* = −0.305, *p* < 0.05). For OV, the expression of IL-17A was positively correlated with the CTL level (GSE31245: *r* = 0.502, *p* < 0.05) and the expression of IL-17C was positively correlated with the CTL level (GSE18521: *r* = 0.433, *p* < 0.05).

The results of survival analysis of the CTL level and expression of IL-17A–F in different tumors are shown in **Figure S9**. Among BRCA, a higher CTL level indicates better patient survival, but only when IL-17A or IL-17C has a high expression level (*p* < 0.05). Among COAD, a lower CTL level indicates better patient survival, but only when IL-25 has a high expression level (*p* < 0.05). Among DLBC, a higher CTL level indicates better patient survival, but only when IL-17A has a high expression level (*p* < 0.05). Among GBM, a higher CTL level indicates better patient survival, but only when IL-17C has a high expression level (*p* < 0.05). Among HNSC, a lower CTL level indicates better patient survival, but only when IL-17A or IL-17F has a high expression level (*p* < 0.05), or IL-25 has a low expression level (*p* < 0.05). Among KIRC, a lower CTL level indicates better patient survival, but only when IL-17C has a high expression level (*p* < 0.05). Among LIHC, a higher CTL level indicates better patient survival, but only when IL-17A or IL-17C has a high expression level (*p* < 0.05), or IL-25 has a low expression level (*p* < 0.05). Among follicular lymphoma, a higher CTL level indicates better patient survival, but only when IL-17C has a high expression level (*p* < 0.05). Among lung cancer (including NSCLC and SCLC), a lower CTL level indicates better patient survival, but only when IL-17D has a high expression level (*p* < 0.05). Among melanoma, a lower CTL level indicates better patient survival, but only when IL-17B or IL-17F has a high expression level (*p* < 0.05). Among NSCLC, a lower CTL level indicates better patient survival, but only when IL-17A has a high expression level (*p* < 0.05), or IL-17C or IL-25 has a low expression level (*p* < 0.05). Among OV, a lower CTL level indicates better patient survival, but only when IL-17B, IL-17C, IL-17D, or IL-17F has a high expression level (*p* < 0.05). Among SARC, a lower CTL level indicates better patient survival, but only when IL-25 has a high expression level (*p* < 0.05). Among STAD, a higher CTL level indicates better patient survival, but only when IL-25 has a high expression level (*p* < 0.05).

### T-cell dysfunction and exclusion analysis

**Figure 6:**
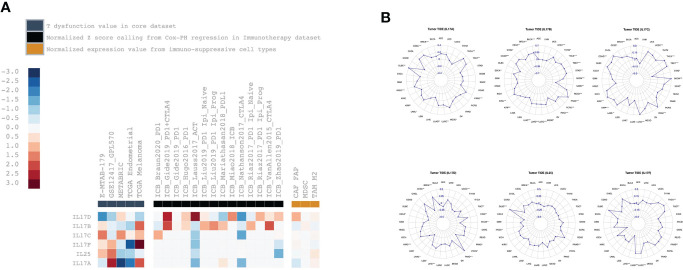
The correlation analysis between IL-17A–F family expression and Tumor Immune Dysfunction and Exclusion-related factors including **(A)** T dysfunction value, normalized *z* score calling from Cox-PH regression in immunotherapy, normalized expression value from immune-suppressive cell types, and **(B)** TIDE values.

The TIDE analysis results are shown in **Figure 6**. For IL-17A, its expression was positively correlated with the T dysfunction value in LAML (GSE12417_GPL570) and melanoma (TCGA), and negatively correlated with the T dysfunction value in BRCA (METABRIC) and UCEC (TCGA); its expression was positively correlated with TIDE value in BRCA (*r* = 0.10, *p* < 0.001), CESC (*r* = 0.27, *p* < 0.001), DLBC (*r* = 0.39, *p* < 0.01), HNSC (*r* = 0.19, *p* < 0.001), PRAD (*r* = 0.21, *p* < 0.001), and THCA (*r* = 0.30, *p* < 0.001). For IL-17B, its expression was positively correlated with the T dysfunction value in melanoma (TCGA); its expression was positively correlated with TIDE value in BLCA (*r* = 0.35, *p* < 0.001), BRCA (*r* = 0.33, *p* < 0.001), COAD (*r* = 0.36, *p* < 0.001), KIRC (*r* = 0.39, *p* < 0.001), LIHC (*r* = 0.19, *p* < 0.001), LUAD (*r* = 0.16, *p* < 0.001), LUSC (*r* = 0.17, *p* < 0.001), PAAD (*r* = 0.31, *p* < 0.001), PCPG (*r* = 0.25, *p* < 0.001), PRAD (*r* = 0.65, *p* < 0.001), READ (*r* = 0.45, *p* < 0.001), STAD (*r* = 0.39, *p* < 0.001), TGCT (*r* = 0.36, *p* < 0.001), THCA (*r* = 0.34, *p* < 0.001), and UCEC (*r* = 0.32, *p* < 0.001). For IL-17C, its expression was positively correlated with the TIDE value in BRCA (*r* = 0.11, *p* < 0.001), COAD (*r* = 0.22, *p* < 0.001), KIRC (*r* = 0.28, *p* < 0.001), KIRP (*r* = 0.20, *p* < 0.001), LUSC (*r* = 0.29, *p* < 0.001), PAAD (*r* = 0.28, *p* < 0.001), SKCM (*r* = 0.29, *p* < 0.001), TGCT (*r* = 0.27, *p* < 0.001), and THCA (*r* = 0.29, *p* < 0.05). For IL-17D, its expression was positively correlated with the normalized *z* score calling from Cox-PH regression in the immunotherapy dataset of Gide2019_PD1+CTLA4 and Lauss2017_ACT; its expression was positively correlated with the TIDE value in BRCA (*r* = 0.28, *p* < 0.001), KIRC (*r* = 0.19, *p* < 0.001), LIHC (*r* = 0.17, *p* < 0.001), and PAAD (*r* = 0.48, *p* < 0.001). For IL-25, its expression was positively correlated with the TIDE value in DLBC (*r* = 0.47, *p* < 0.001) and PRAD (*r* = 0.24, *p* < 0.001). For IL-17F, its expression was positively correlated with T dysfunction value in melanoma (TCGA), and negatively correlated with the T dysfunction value in UCEC (TCGA); its expression was positively correlated with the TIDE value in LUAD (*r* = 0.19, *p* < 0.001), LUSC (*r* = 0.19, *p* < 0.001), and THCA (*r* = 0.23, *p* < 0.001), and negatively correlated with the TIDE value in LIHC (*r* = −0.18, *p* < 0.001).

### Immunomodulator expression analysis

**Figure 7:**
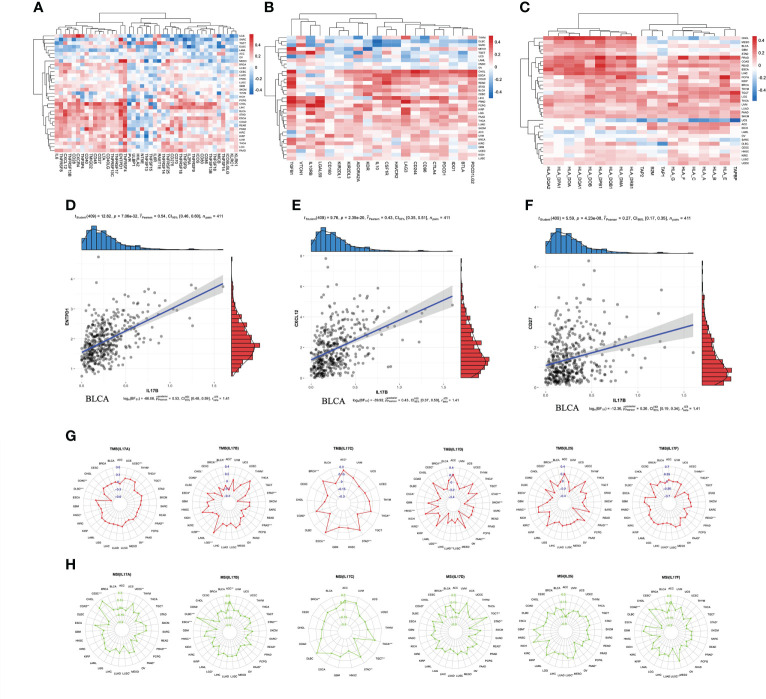
The correlation between IL-17B expression and immunomodulators including immunostimulators **(A)**, immunoinhibitors **(B)**, and MHC molecules **(C)**. The scatter plots represent the top four strongest correlations in each heatmap **(D–F)**. The correlation between IL-17A–F expression and immunotherapeutic biomarker (TMB and MSI). The red radar charts represent the association between TMB **(G)** and IL-17A, IL-17B, IL-17C, IL-17D, IL-25, and IL-17F. The green radar charts represent MSI **(H)** and IL-17A, IL-17B, IL-17C, IL-17D, IL-25, and IL-17F. (∗∗∗*p* < 0.001; ∗∗*p* < 0.01; ∗*p* < 0.05.).

Immunomodulators can be further divided into immunoinhibitors, immunostimulators, and major histocompatibility complex (MHC) molecules. Spearman correlation between IL-17B expression and immunomodulators was analyzed using the TISIDB database (**Figure 7**). **Figure 7A** shows the correlation between IL-17B expression level in pan-cancer and immunostimulators. The strongest correlations of ENTPD1 (*r* = 0.54, *p* < 0.001), CXCL12 (*r* = 0.43, *p* < 0.001), CD27 (*r* = 0.27, *p* < 0.001), and IL-17B were in BLCA (**Figures 7D–F**). **Figure 7B** shows the correlation between IL-17B expression level and immunoinhibitors. In some tumors, such as CHOL, ESCA, and COAD, there were mainly positive correlations. In other tumors, such as DLBC and SRAC, there were mainly negative correlations. **Figure 7C** shows the correlation between IL-17B expression level and MHC molecules. There were mainly positive correlations in most tumors except UCS.

We further explored the correlation between IL-17A–F and ICI biomarkers (TMB and MSI) (**Figure 7**). The expression of IL-17A was positively correlated with TMB values in UCEC (*R* = 0.19, *p* < 0.001), and negatively correlated with TMB values in DLBC (*r* = −0.52, *p* < 0.001). The expression of IL-17B was positively correlated with TMB values in LGG (*r* = 0.30, *p* < 0.001), and negatively correlated with TMB values in BRCA (*r* = −0.52, *p* < 0.001), PRAD (*r* = −0.32, *p* < 0.001), and STAD (*r* = −0.35, *p* < 0.001). The expression of IL-17C was positively correlated with TMB values in BRCA (*r* = 0.17, *p* < 0.001) and STAD (*r* = 0.19, *p* < 0.001). The expression of IL-17D was negatively correlated with TMB values in BRCA (*r* = −0.15, *p* < 0.001), HSNC (*r* = −0.18, *p* < 0.001), LGG (*r* = −0.32, *p* < 0.001), LUAD (*r* = −0.17, *p* < 0.001), PAAD (*r* = −0.36, *p* < 0.001), SKCM (*r* = −0.29, *p* < 0.001), and STAD (*r* = −0.19, *p* < 0.001). The expression of IL-25 was negatively correlated with TMB values in PRAD (*r* = −0.34, *p* < 0.001). The expression of IL-17F was negatively correlated with TMB values in READ (*r* = −0.28, *p* < 0.001) and THYM (*r* = −0.63, *p* < 0.001). The expression of IL-17A was positively correlated with MSI values in CESC (*r* = 0.25, *p* < 0.001), and negatively correlated with MSI values in PRAD (*r* = −0.16, *p* < 0.001). The expression of IL-17B was positively correlated with MSI values in DLBC (*r* = 0.46, *p* < 0.001), and negatively correlated with MSI values in ESCA (*r* = −0.29, *p* < 0.001) and STAD (*r* = −0.22, *p* < 0.001). The expression of IL-17C was positively correlated with MSI values in THCA (*r* = 0.18, *p* < 0.001). The expression of IL-17F was negatively correlated with MSI values in COAD (*r* = −0.21, *p* < 0.001).

### GSEA

**Figure 8:**
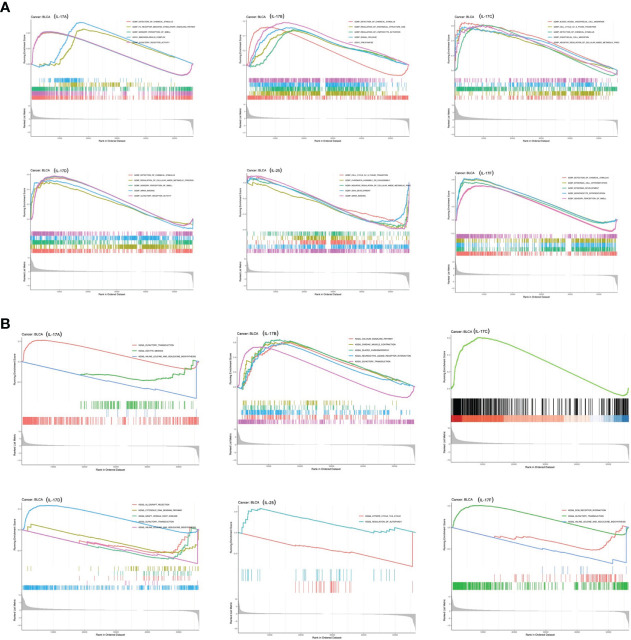
GSEA based on GO **(A)** and KEGG **(B)** databases between the IL-17A–F low-expression group and high-expression group in BLCA.

GSEA was applied to identify signaling pathways involved in tumorigenesis of BLCA between low and high IL-17 family gene expression in the KEGG and GO databases (**Figure 8**). It was obvious that significant differences existed in the enrichment of both GO and KEGG pathways in high IL-17 member expression groups. It showed that pathways including immunoglobulin complex, detection of chemical stimulus, regulation of lymphocyte activation, signal release, cell cycle G1_S phase transition, detection of chemical stimulus, endothelial cell migration, and negative regulation of cellular amide metabolic process were significantly differentially enriched in IL-17 high-expression groups.

### Immunotherapeutic response analysis

**Figure 9:**
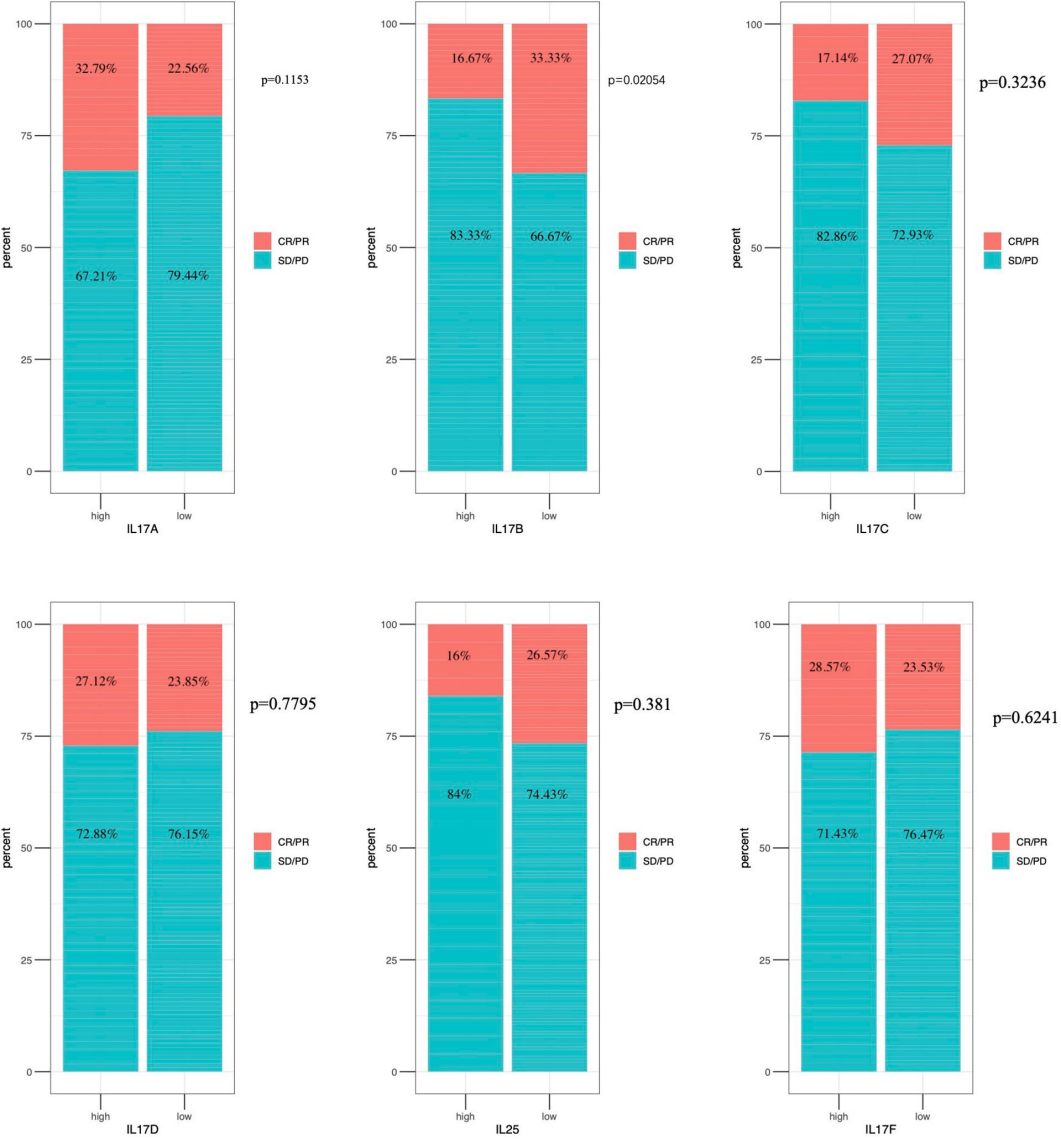
Immunotherapeutic response analysis. The differential expression of IL-17A–F between non-response and response in IMvigor 210.

**Figure 10:**
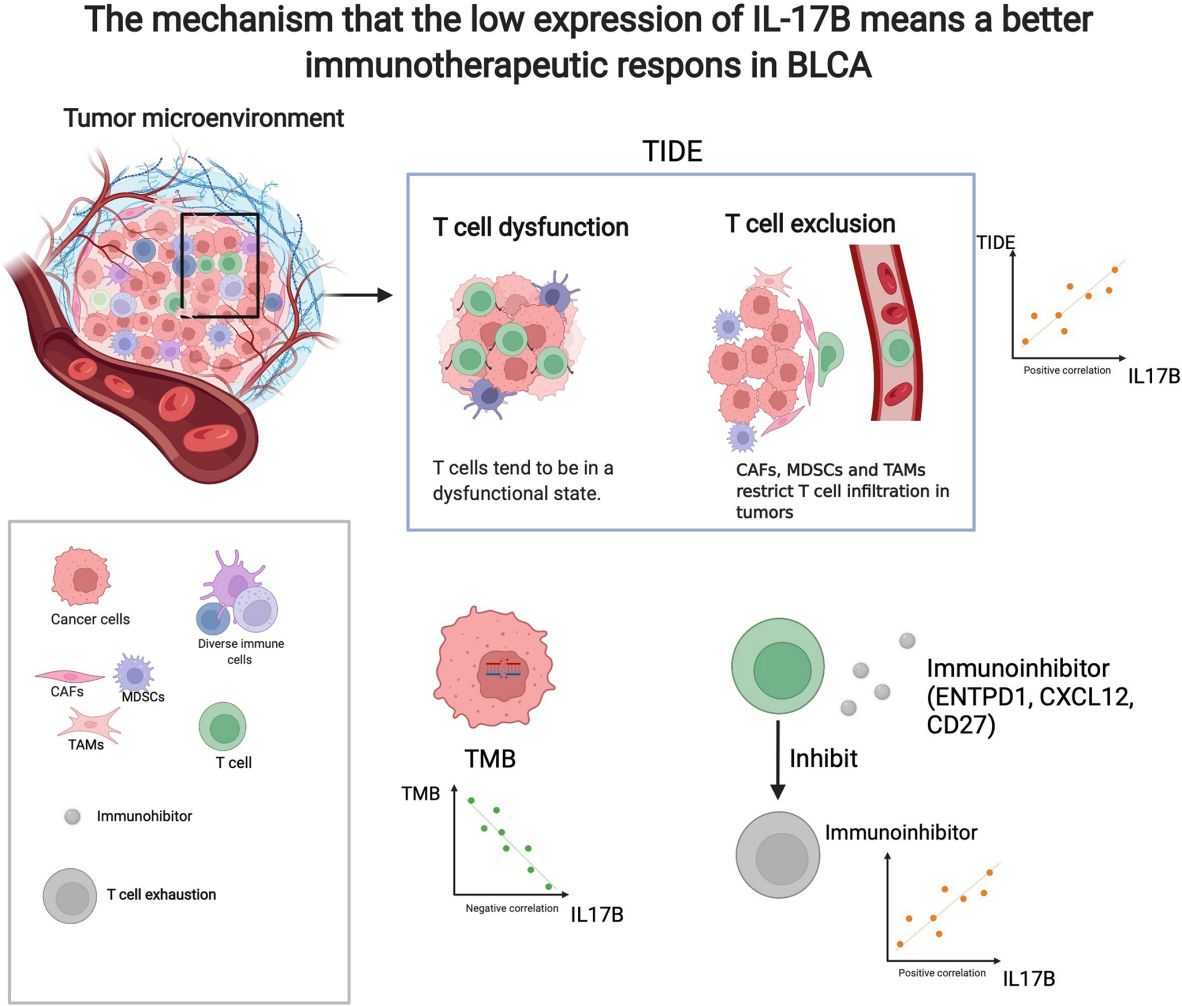
The mechanism that the low expression of IL-17B means a better immunotherapeutic response in BLCA. (created with biorender.com).

As shown in **Figure 9**, there was significant difference in IL-17B expression (*p* < 0.05) between responders and non-responder groups or between the PD+SD group and the CR+PR group; there was no significant difference in IL-17A, IL-17C, IL-17D, IL-25, and IL-17F expression between responders and non-responder groups or between the PD+SD group and the CR+PR group. The mechanism that the low expression of IL-17B means a better immunotherapeutic response in BLCA is summarized in **Figure 10**, which has been described above.

### Drug sensitivity analysis in pan-cancer

**Figure S10** shows the correlation between drug sensitivity and IL-17A–F genes’ mRNA expression. Blue bubbles represent negative correlations, and red bubbles represent positive correlations; the deeper the color, the higher the correlation. Bubble size is positively correlated with the FDR significance. Black outline border indicates FDR ≤ 0.05. There was a negative correlation between expression of IL-17A and drug sensitivity including BI-2536, BRD-K51490254, BRD-K61166597, BRD-K66453893, and BRD-K70511574. The expression of IL-17B was negatively correlated with drug sensitivity including BRD-K35604418, BRD-K41597374, BRD1812, and CIL55A. The expression of IL-17C was positively correlated with drug sensitivity including 1S,3R-RSL-3, AT7867, and AZD4547. The expression of IL-17D was negatively correlated with drug sensitivity including AZD4547, BIX-01294, BMS-754807, and BRD-A94377914. The expression of IL-25 was negatively correlated with drug sensitivity including BMS-345541, BRD-K66532283, COL-3, and CR-1-31B. The expression of IL-17F was negatively correlated with drug sensitivity including B02, BI-2536, BMS-345541, and BRD-K66453893.

## Discussion

In our present research, we performed analysis including differential analysis, coexpression analysis, gene activity analysis, clinical analysis, immune subtype analysis, stemness analysis, TME score analysis, immune correlation analysis, and drug sensitivity to explore the correlation between the gene expression level of the IL-17 family and indicators of diagnosis, prognosis, and treatment of patients. From the multiple analysis, we obtained the conclusion that IL-17B might act as a biomarker of ICI response of patients with BLCA.

In the TCGA dataset, we compared the differential expression between tumor samples and adjacent tissue samples and found that IL-17 expressed differently in different tumor types. By comparing the transcription level with IL-17 gene activity score, the transcription level of IL-17 in most cancers partially matched the overall IL-17 activation, indicating that the transcriptional level represented IL-17 gene activation in these cancers. However, in some tumors (LIHC and CHOL), inconsistencies between IL-17B and IL-17D expression and activity were observed, and this may be caused by the regulation of transcription and translation. We conducted survival analysis and different clinical stages analysis to judge the patient’s prognosis and the progression of tumor. From Kaplan–Meier plots, we could obtain that the expression of IL-17 has a certain influence on the prognosis of patients; taking COAD as an example, patients with high IL-17A expression had a better survival rate than patients with low IL-17A expression, indicating that IL-17 can be a factor predicting tumor prognosis. As for the result of the stage analysis, IL-17B had statistical significance in BRCA, and the low-expression group predicted tumor progression and poor patient prognosis. We hypothesized that therapeutic modulation of IL-17 activity in various tumor types may be an effective strategy with clinical benefit.

In recent years, cancer stemness has become an important feature of cancer (63). Emerging evidence suggests that cancer stem cells (CSCs) are a subpopulation of cells with stem cell-like characteristics that are essential for the occurrence, progression, recurrence, metastasis, and chemoresistance of cancers (64–66). Here, we used the OCLR method to calculate the RNAss scores and DNAss scores, which were two types of stemness indices of tumor samples, and then correlated them with the transcription characteristics of IL-17. For example, the expression of IL-17B was strongly negatively associated with DNAss and RNAss in PCPG, suggesting that high expression of IL-17B may lead to low stemness of tumor in PCPG.

TME includes non-malignant cells, blood vessels, lymphoid organs or lymph nodes, nerves, intercellular components, and metabolites located in the center, margins, or near the tumor focus (67). The six stable and reproducible immune subtypes C1–C6 cover almost all human malignancies. These subtypes are related to changes in prognosis, genetics, and immune regulation, and represent the characteristics of TME (26). In our results, it is confirmed that the expression of IL-17A, IL-17B, IL-17C, IL-17D, IL-17E (IL-25), and IL-17F in tumor immune subtypes is statistically significant, especially in BRCA; there may be a potential microenvironmental regulation mechanism that remains to be investigated. Different from tumor cells, stromal cells are genetically stable in the TME and therefore represent an attractive therapeutic target to reduce drug resistance and the risk of tumor recurrence (62). In order to analyze the relationship between TME and the IL-17 family in more detail to facilitate an in-depth study of the clinical treatment plan of tumors, we calculated stromal scores, immune scores, and ESTIMATE scores, and analyzed the relationship between IL-17 genes’ expression level and these three scores through Spearman correlation. Not surprisingly, these TME features were positively related to the expression level of IL-17B remarkably. Research has confirmed that TME plays a crucial role in the intrinsic and acquired resistance of ICIs, and understanding original resistance may help discover predictive biomarkers (68).

In order to further study the role of IL-17 on tumor immunotherapy, in-depth research related to immunotherapy was conducted. Here, we focused on the role of IL-17B as a predictive biomarker in immunotherapy. The expression of IL-17B in pan-cancer has a significantly positive correlation with CAF, Endo, HSC, and NKT, showing that the highly expressed IL-17B may have higher immune infiltration of these cells in TME. Parisa et al. found that CAF passed through the TGF-β signal pathway to inhibit antitumor immune response (69). Thus, high IL-17B may contribute to tumor immune exclusion. In terms of TIDE, the expression of IL-17B was also positively correlated with the TIDE values in many tumors such as BRCA, BLCA, KIRC, and PRAD. A higher tumor TIDE prediction score is associated not only with worse ICB response, but also with worse patient survival under anti-PD1 and anti-CTLA4 therapies (37). Thus, high IL-17B may be associated with worse ICB response and worse patient survival in these tumors. TMB and MSI have also long been considered as biomarkers of ICI response and played an important role in tumors (70, 71). Our study showed that IL-17B expression was negatively associated with TMB in BLCA, ACC, BRCA, and STAD and negatively associated with MSI in ESCA, STAD, and PAAD, suggesting that these tumors with low IL-17B expression may be beneficial to a better response rate in ICI immunotherapy. The above results were consistently correlated with the immunotherapy results of the IMvigor cohort.

Interestingly, we found that the expression of IL-17B was positively correlated with most immune checkpoints in some tumors, such as CHOL and BLCA. ICIs such as anti-PDL1/PD1 and/or CTLA4 antigen have become more and more popular, but only patients with high expression of immune checkpoint genes are more sensitive to treatment (72). Strikingly, GSEA showed that pathways including regulation of lymphocyte activation imply that the high expression of IL-17 might be associated with immune response of some cancers, but more details need to be studied. This study also found that the members of IL-17 are associated with drug sensitivity. This suggests that it may be possible to select antitumor drugs through the detection of tumor genes.

To the best of our knowledge, this is the first study to investigate the values of the IL-17 family in pan-cancer systematically. This study reveals various expression forms of the IL-17 family in the pan-cancer range, and the expression of IL-17 family genes might act as a biomarker in the prediction of immunotherapy (ICIs) effects. Although this experiment confirmed that IL-17 played an important role in pan-cancer, it still has shortcomings. This research was mainly based on databases, lacking more comprehensive animal experiments and other verification.

## Conclusion

We applied multiple analyses on IL-17A, IL-17B, IL-17C, IL-17D, IL-17E (IL-25), and IL-17F, including differential expression analysis, coexpression analysis, gene activity analysis, clinical analysis, immune subtype analysis, stemness analysis, TME score analysis, immunotherapeutic correlation analysis, and drug sensitivity. Collectively, IL-17 family members may act as biomarkers in predicting the prognosis of the tumor and the therapeutic effects of ICIs, which provides new guidance for cancer treatment. However, considering the use of bioinformatics methods, the current results are preliminary and further verification is required.

## Data availability statement

The datasets presented in this study can be found in online repositories. The names of the repository/repositories and accession number(s) can be found in the article/**Supplementary Material**.

## Ethics statement

Written informed consent was obtained from the individual(s) for the publication of any potentially identifiable images or data included in this article.

## Author contributions

XH designed the manuscript. JY wrote the manuscript, and XH revised and completed the manuscript. TM, JL, RH, and DS revised the manuscript. XH downloaded and analyzed data. DS sponsored and provided the funding. All authors contributed to the article and approved the submitted version.

## Acknowledgments

We would like to thank The Cancer Genome Atlas (TCGA), the Gene Expression Omnibus (GEO) database (https://www.ncbi.nlm.nih.gov/geo/), the STRING database (https://string-db.org/), the Human Protein Atlas (https://www.proteinatlas.org/), Genomics of Drug Sensitivity in Cancer (GDSC) (https://www.cancerrxgene.org/), the Cancer Therapeutics Response Portal (CTRP) (https://portals.broadinstitute.org/ctrp/), and the METABRIC database for using their data and firebrowse (http://firebrowse.org/), Gene Set Cancer Analysis (GSCA) (http://bioinfo.life.hust.edu.cn/GSCA/#/immune), the TISIDB database (http://cis.hku.hk/TISIDB), TIMER2.0 (http://timer.cistrome.org/), UCSC Xena (http://xena.ucsc.edu/), Tumor Immune Dysfunction and Exclusion (TIDE: http://tide.dfci.harvard.edu), hipplot (hiplot.com.cn) and Cytoscape (Version: 3.8.2) for their help in analyzing the data, thank to biorender (https://biorender.com/) for its help in making mechanism diagram diagram, thank to R (Version: 4.1.1) and people who offer the R package used in our manuscript. We also appreciate Zihao Chen (University Hospital for Gynecology, Pius-Hospital, University Medicine Oldenburg, Oldenburg, Germany) and Jiayu Liu (School of Mechanical Engineering, Shanghai Jiao Tong University, Shanghai 200201, China) for their help in analyzing data. This literature was supported by the National Natural Science Foundation of China (82073207).

## Conflict of interest

The authors declare that the research was conducted in the absence of any commercial or financial relationships that could be construed as a potential conflict of interest.

## Publisher’s note

All claims expressed in this article are solely those of the authors and do not necessarily represent those of their affiliated organizations, or those of the publisher, the editors and the reviewers. Any product that may be evaluated in this article, or claim that may be made by its manufacturer, is not guaranteed or endorsed by the publisher.
