# Supplementary material for: Pan-cancer analysis reveals interleukin-17 family members as biomarkers in the prediction for immune checkpoint inhibitor curative effect
Source: Front Immunol. 2022 Sep 8;13:900273. doi: 10.3389/fimmu.2022.900273 (PMC9493092; doi:10.3389/fimmu.2022.900273)
Supplement: Supplementary file 1 [file DataSheet_1.zip › Supplementary materials/Fig.S3/UVM.pdf]

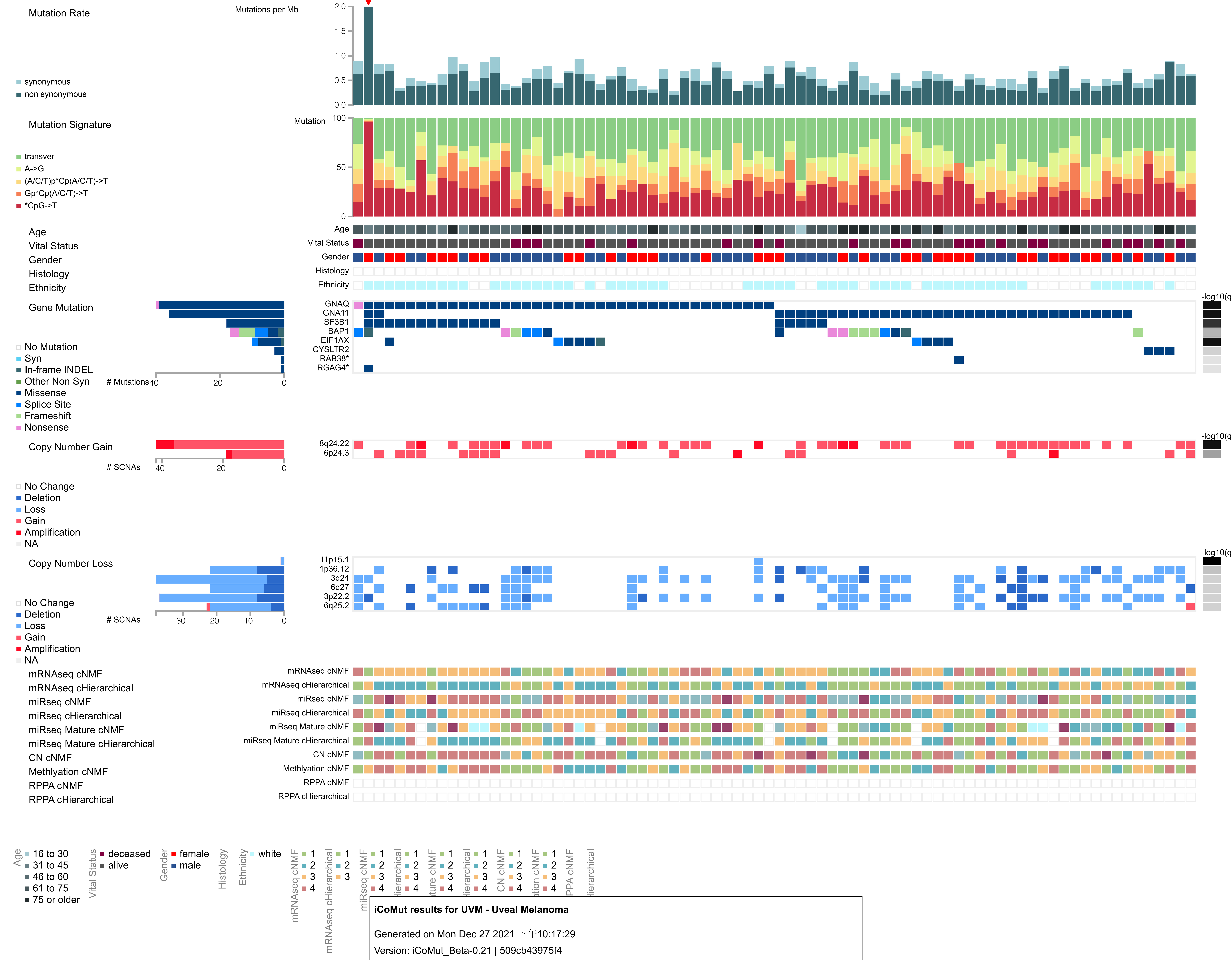

iCoMut results for UVM - Uveal Melanoma

Generated on Mon Dec 27 2021 下午10:17:29

Version: iCoMut_Beta-0.21 | 509cb43975f4

View on Firebrowse at:

http://firebrowse.org/iCoMut/?cohort=UVM
