# Supplementary material for: Pan-cancer analysis reveals interleukin-17 family members as biomarkers in the prediction for immune checkpoint inhibitor curative effect
Source: Front Immunol. 2022 Sep 8;13:900273. doi: 10.3389/fimmu.2022.900273 (PMC9493092; doi:10.3389/fimmu.2022.900273)
Supplement: Supplementary file 1 [file DataSheet_1.zip › Supplementary materials/Table S1.docx]

**Supplementary Table S1. Abbreviations and full names of nouns in the text**

| Shorthand | Full name |
| --- | --- |
| ACC | Adrenocortical carcinoma |
| BLCA | Bladder Urothelial Carcinoma |
| BRCA | Breast invasive carcinoma |
| CESC | Cervical squamous cell carcinoma and endocervical adenocarcinoma |
| CHOL | Cholangiocarcinoma |
| COAD | Colon adenocarcinoma |
| DLBC | Lymphoid Neoplasm Diffuse Large B-cell Lymphoma |
| ESCA | Esophageal carcinoma |
| GBM | Glioblastoma multiforme |
| HNSC | Head and Neck squamous cell carcinoma |
| KICH | Kidney Chromophobe |
| KIRC | Kidney renal clear cell carcinoma |
| KIRP | Kidney renal papillary cell carcinoma |
| LAML | Acute Myeloid Leukemia |
| LGG | Brain Lower Grade Glioma |
| LIHC | Liver hepatocellular carcinoma |
| LUAD | Lung adenocarcinoma |
| LUSC | Lung squamous cell carcinoma |
| MESO | Mesothelioma |
| OV | Ovarian serous cystadenocarcinoma |
| PAAD | Pancreatic adenocarcinoma |
| PCPG | Pheochromocytoma and Paraganglioma |
| PRAD | Prostate adenocarcinoma |
| READ | Rectum adenocarcinoma |
| SARC | Sarcoma |
| SKCM | Skin Cutaneous Melanoma |
| STAD | Stomach adenocarcinoma |
| TGCT | Testicular Germ Cell Tumors |
| THCA | Thyroid carcinoma |
| THYM | Thymoma |
| UCEC | Uterine Corpus Endometrial Carcinoma |
| USC | Uterine Carcinosarcoma |
| UVM | Uveal Melanoma |
| IL-17 | Interleukin 17 |
| ICIs | Immune checkpoint inhibitors |
| TCGA | The Cancer Genome Altas |
| TME | Tumor microenvironment |
| PDAC | Pancreatic ductal adenocarcinoma |
| CTLA-4 | Cytotoxic T lymphocyte antigen 4 |
| PD-1 | Programmed death 1 |
| PD-L1 | Programmed death-ligand 1 |
| OS | Overall survival |
| TIDE | Tumor Immune Dysfunction and Exclusion |
| TMB | Tumor mutational burden |
| MSI | Microsatellite instability |
| PPI | Protein-protein interaction |
| ssGSEA | Single sample gene set enrichment analysis |
| DNAss | DNA methylation-based stemness index |
| RNAss | mRNA expression-based stemness index |
| ICB | immune checkpoint blockers |
| CTL | Cytotoxic T lymphocytes |
| CAFs | Cancer-associated fibroblasts |
| MDSCs | Myeloid-derived suppressor cells |
| TAMs | Tumor-associated macrophages |
| CR | Complete remission |
| PR | Partial remission |
| PD | Progressive disease |
| SD | Stable disease |
| GDSC | Genomics of Drug Sensitivity in Cancer |
| CTRP | Cancer Therapeutics Response Portal |
| MHC | Major histocompatibility complex |
| CSC | Cancer stem cells |
