# Supplementary material for: Pan-cancer analysis reveals interleukin-17 family members as biomarkers in the prediction for immune checkpoint inhibitor curative effect
Source: Front Immunol. 2022 Sep 8;13:900273. doi: 10.3389/fimmu.2022.900273 (PMC9493092; doi:10.3389/fimmu.2022.900273)
Supplement: Supplementary file 1 [file DataSheet_1.zip › Supplementary materials/Table S2.docx]

**Supplementary Table S2. Primer information**

| **Primer**  **Symbol** | **Gene name** | **Primer**  **direction** | **Sequences (5’to 3’)** |
| --- | --- | --- | --- |
| IL-17B | Interleukin 17B | Forward  Reverse | GCTGTGGATGTCCAACAAGAGG  TCCTGCATGGTGAAGGGGTTCA |
| IL-17C | Interleukin 17C | Forward  Reverse | GCCCTCAGCTACGACCCAGTG  AGCTTCTGTGGATAGCGGTCCT |
| IL-17D | Interleukin 17D | Forward  Reverse | CTGAATATGAGGACAAAGTGGGC  ACCTAGCTCTCGTGGCAGAATG |
| IL-25 | Interleukin 25 | Forward  Reverse | AACCGCCACCCAGAGTCCTGT  ACAGGCAACGGGCGTGGTACA |
| β-actin | Beta-actin | Forward  Reverse | CACCATTGGCAATGAGCGGTTC  AGGTCTTTGCGGATGTCCACGT |
