# Supplementary figures and images for: Pan-cancer analysis reveals interleukin-17 family members as biomarkers in the prediction for immune checkpoint inhibitor curative effect
Source: Front Immunol. 2022 Sep 8;13:900273. doi: 10.3389/fimmu.2022.900273 (PMC9493092; doi:10.3389/fimmu.2022.900273)

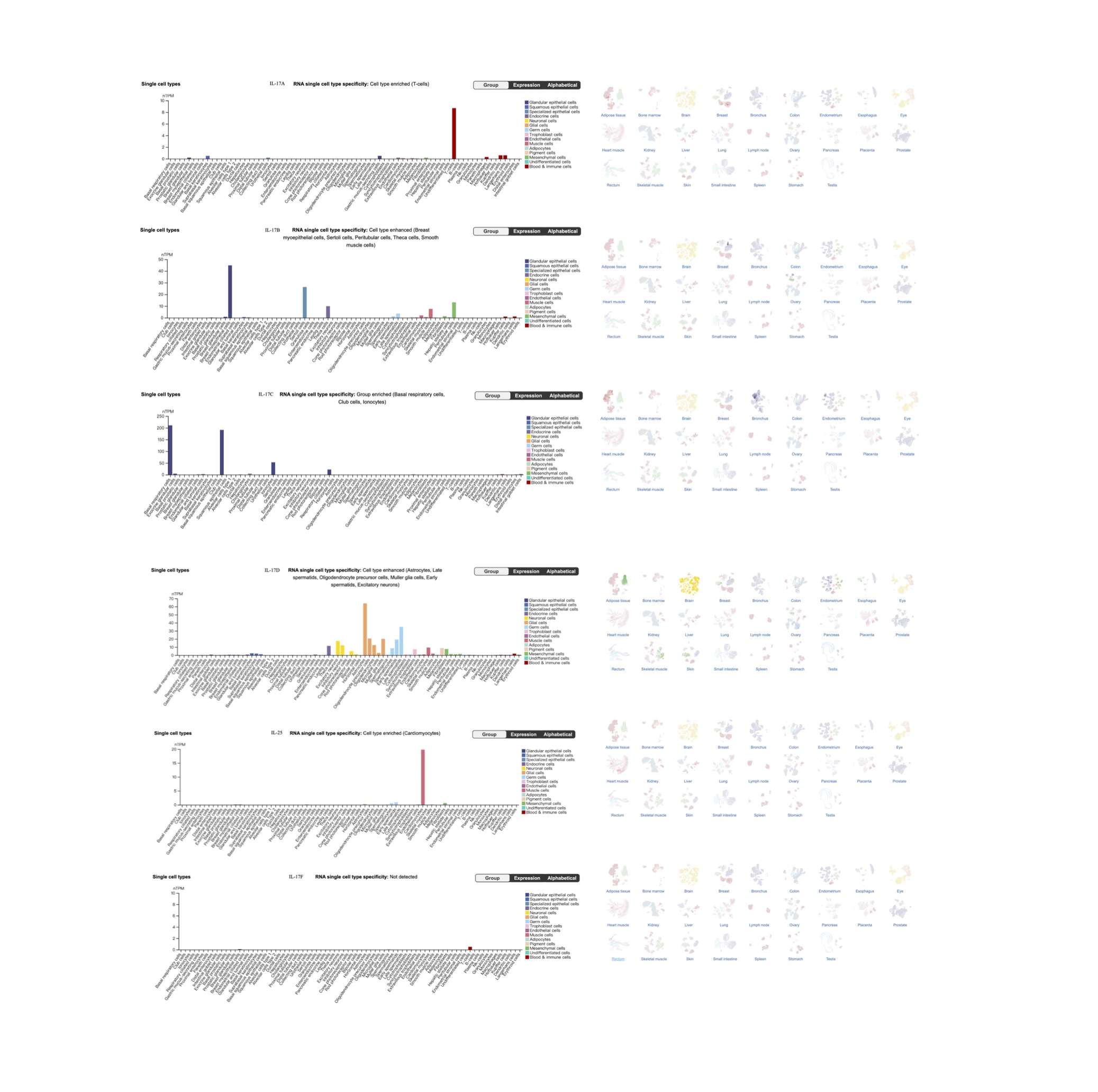

Supplement: Supplementary file 1 [file DataSheet_1.zip › Supplementary materials/Fig.S1.jpg]

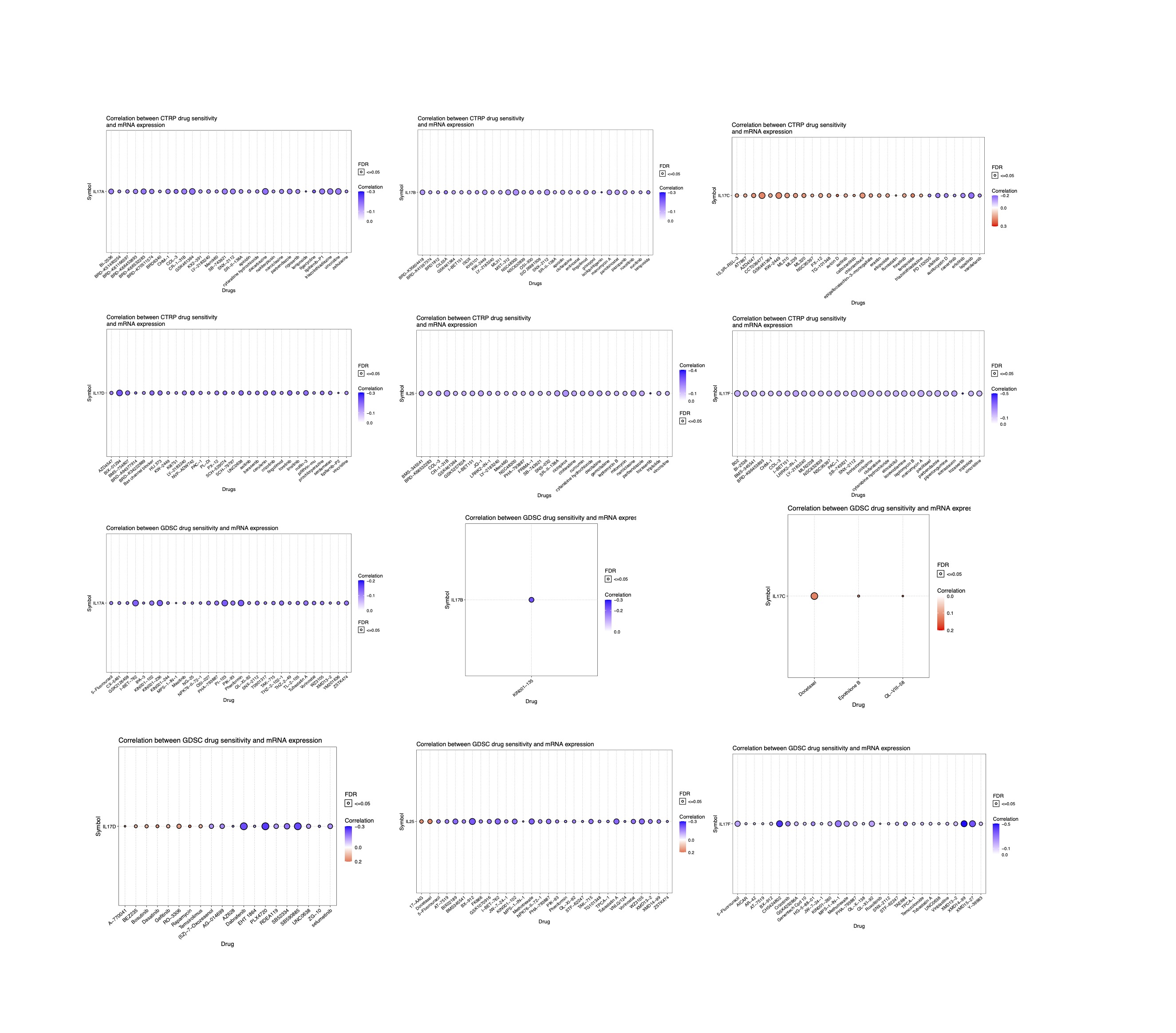

Supplement: Supplementary file 1 [file DataSheet_1.zip › Supplementary materials/Fig.S10 .jpg]

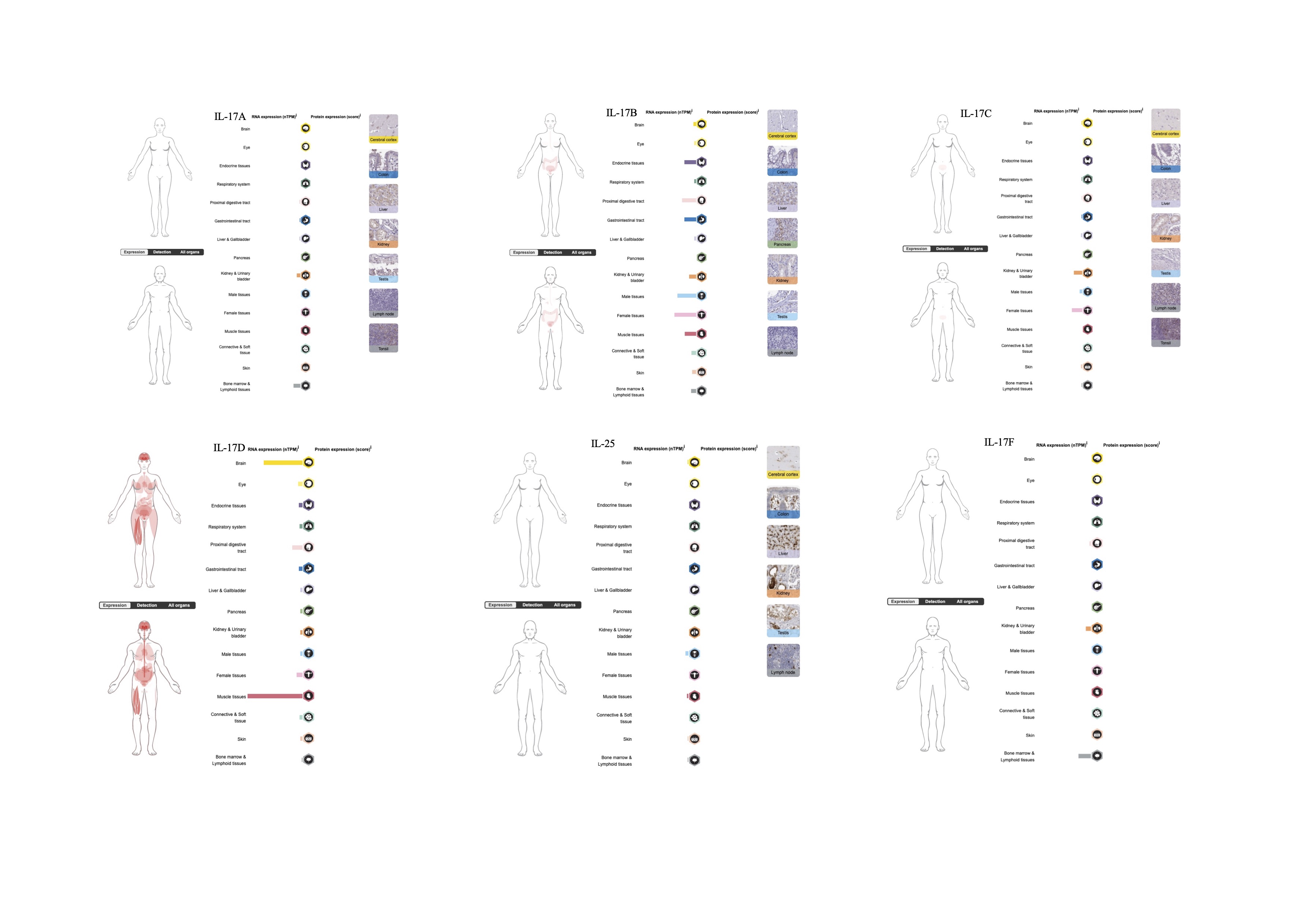

Supplement: Supplementary file 1 [file DataSheet_1.zip › Supplementary materials/Fig.S2.jpg]

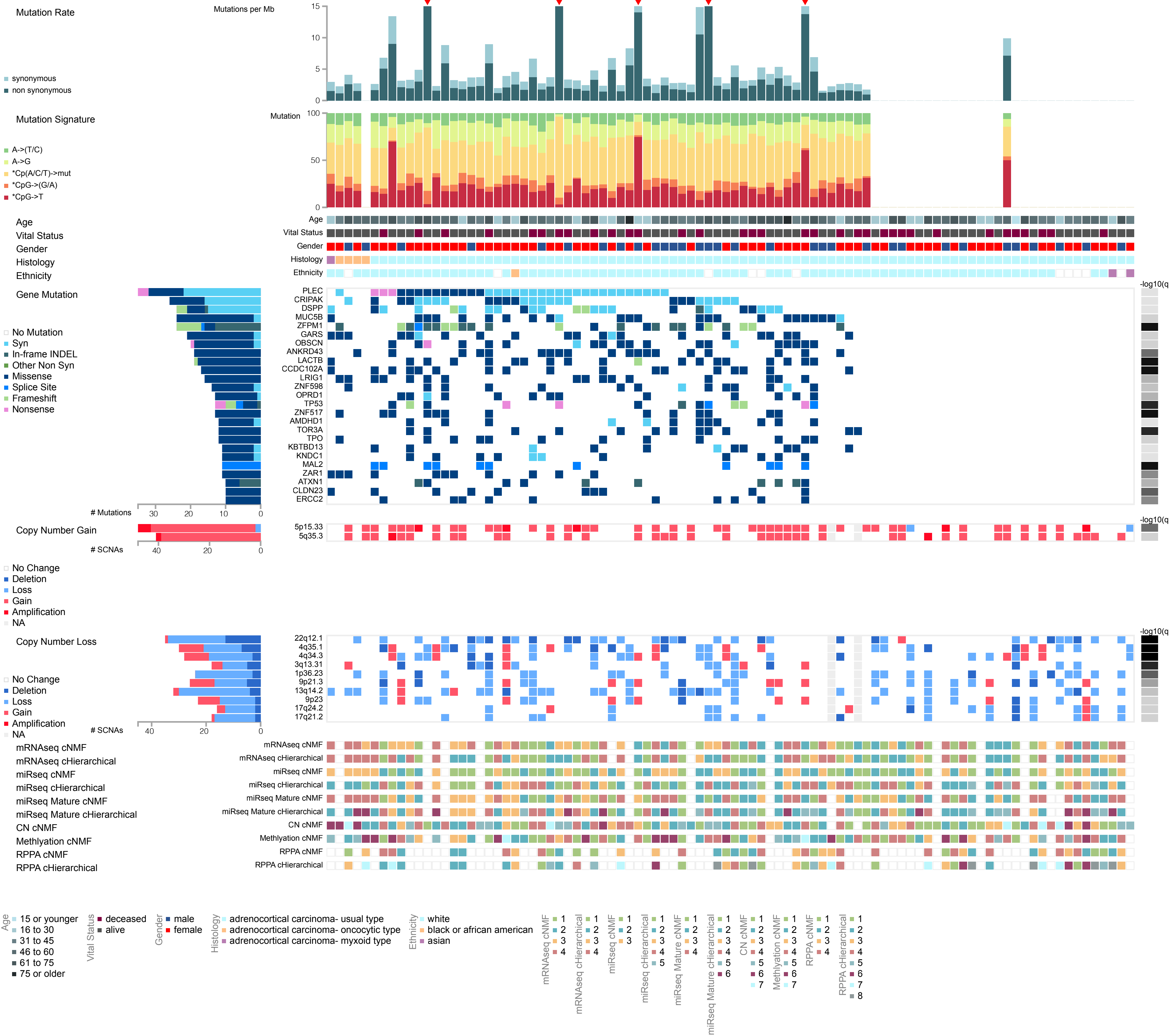

Supplement: Supplementary file 1 [file DataSheet_1.zip › Supplementary materials/Fig.S3/ACC.pdf]

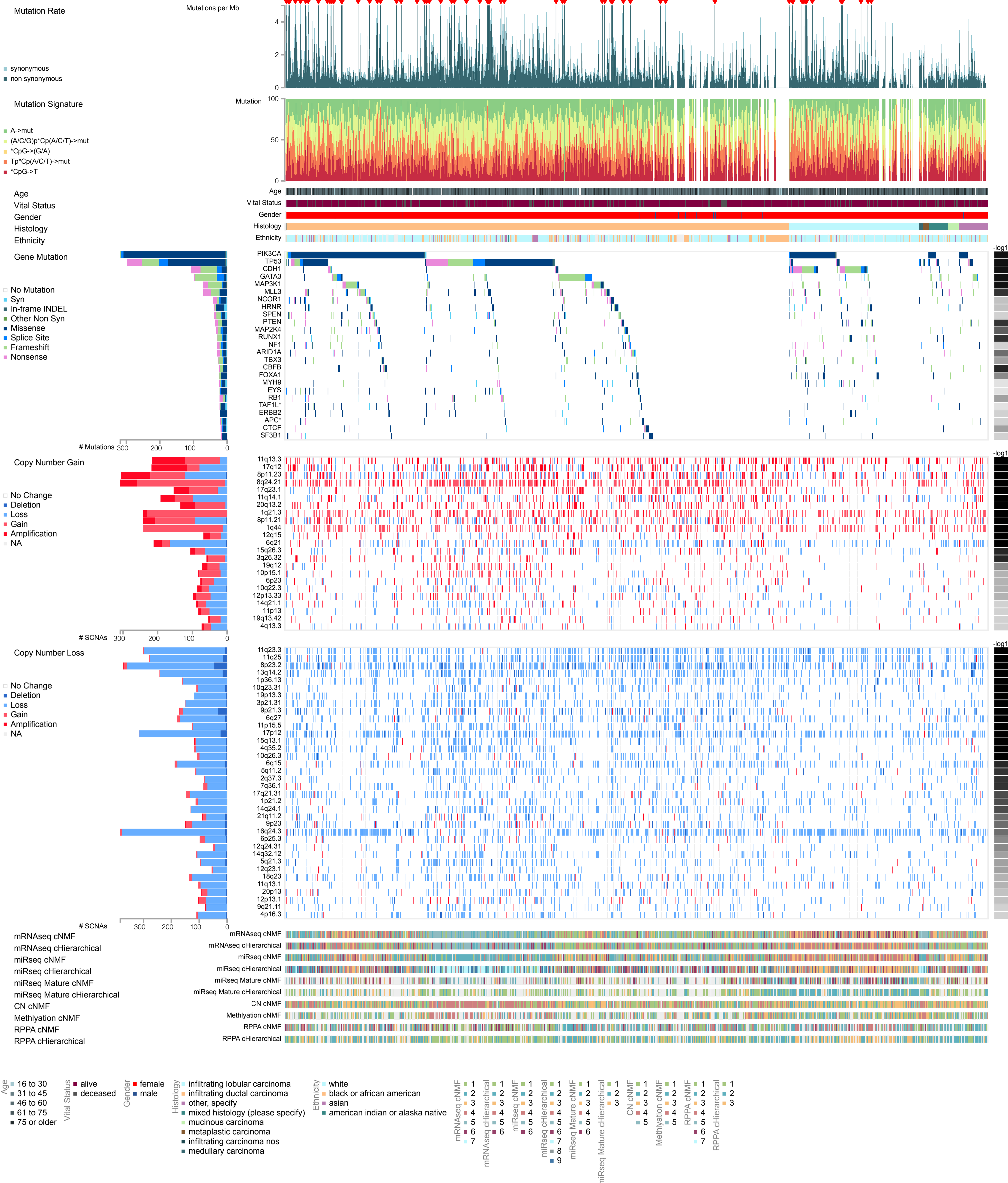

Supplement: Supplementary file 1 [file DataSheet_1.zip › Supplementary materials/Fig.S3/BRCA.pdf]

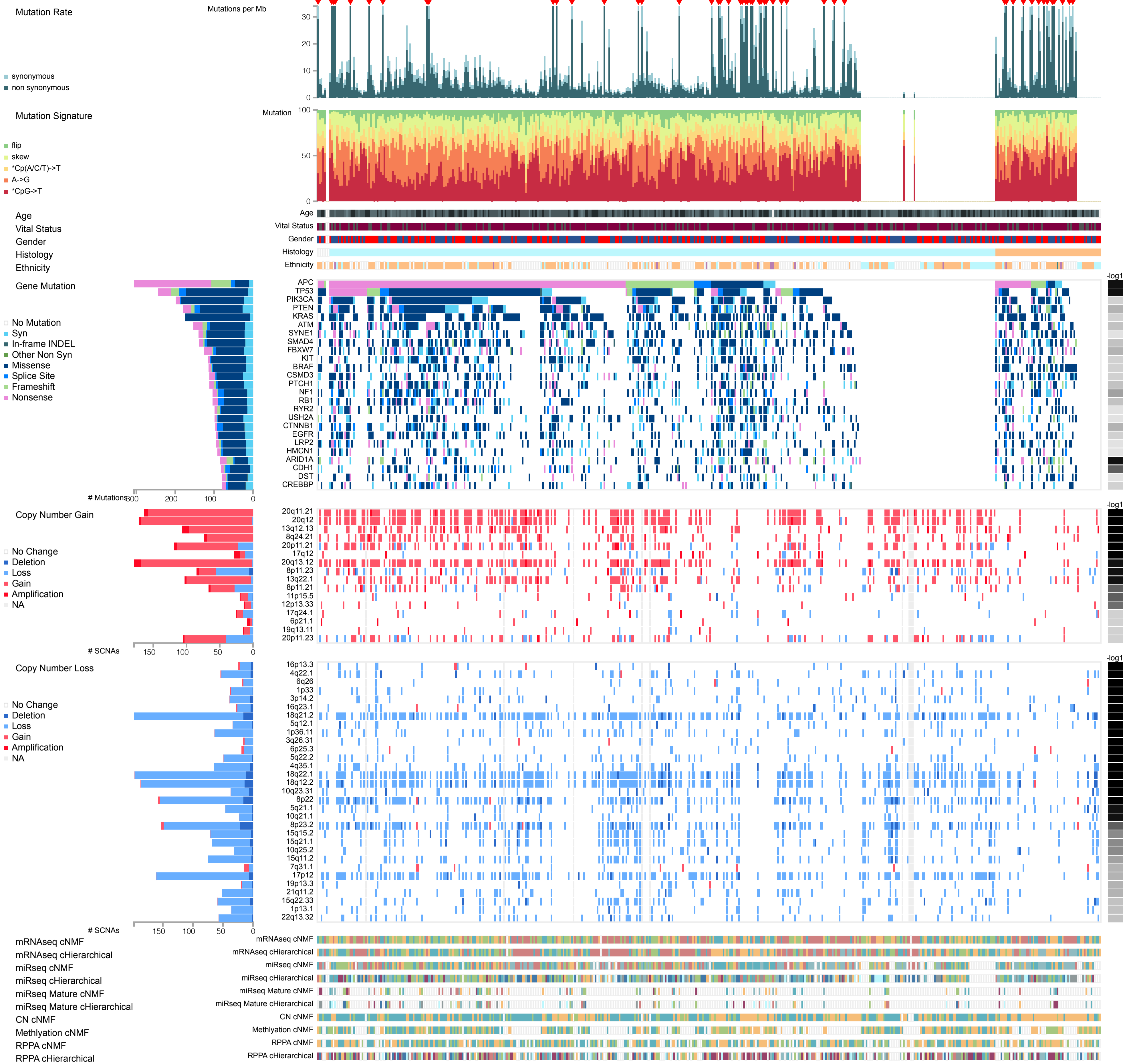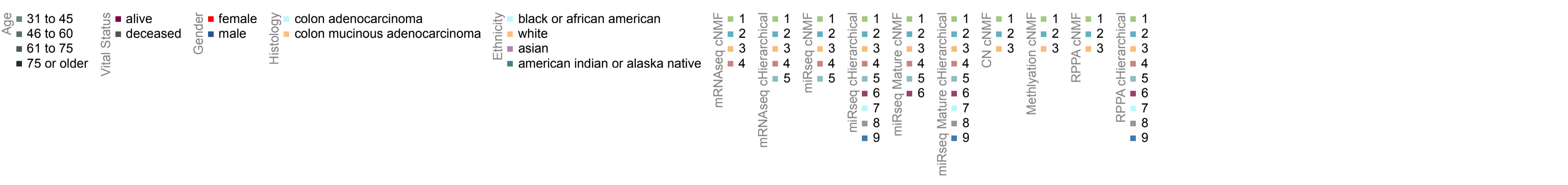

Supplement: Supplementary file 1 [file DataSheet_1.zip › Supplementary materials/Fig.S3/COAD.pdf]

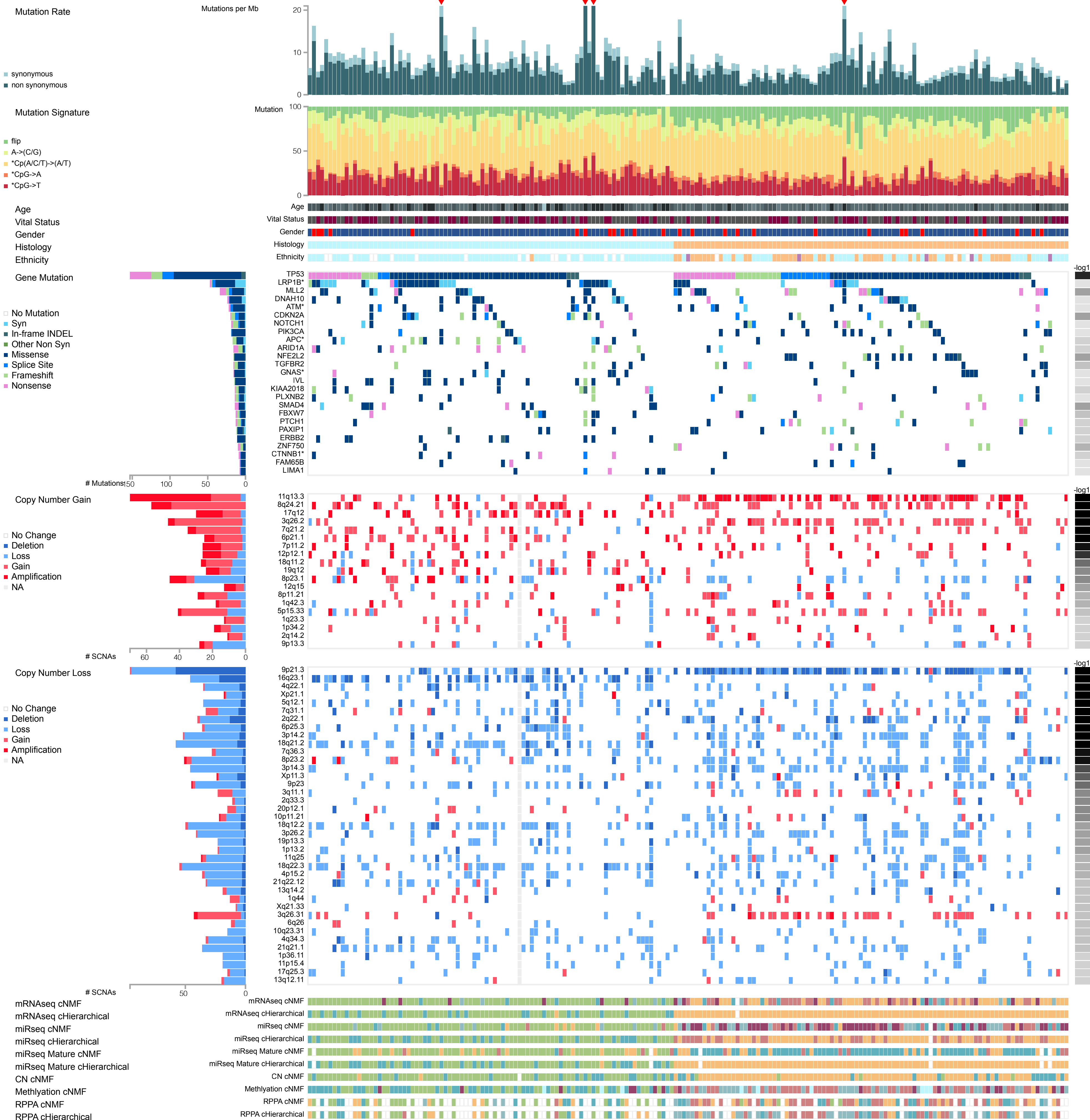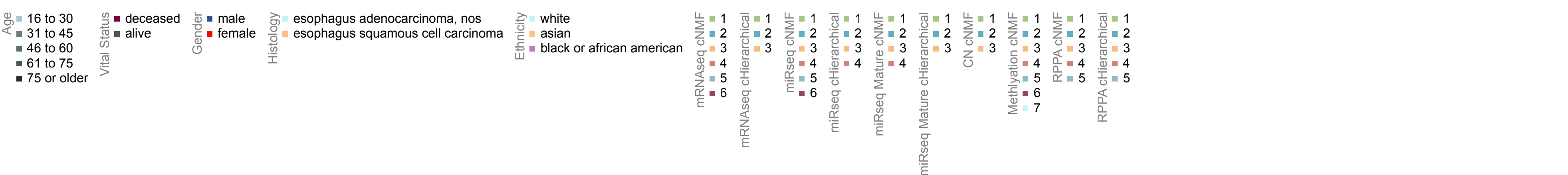

Supplement: Supplementary file 1 [file DataSheet_1.zip › Supplementary materials/Fig.S3/ESCA.pdf]

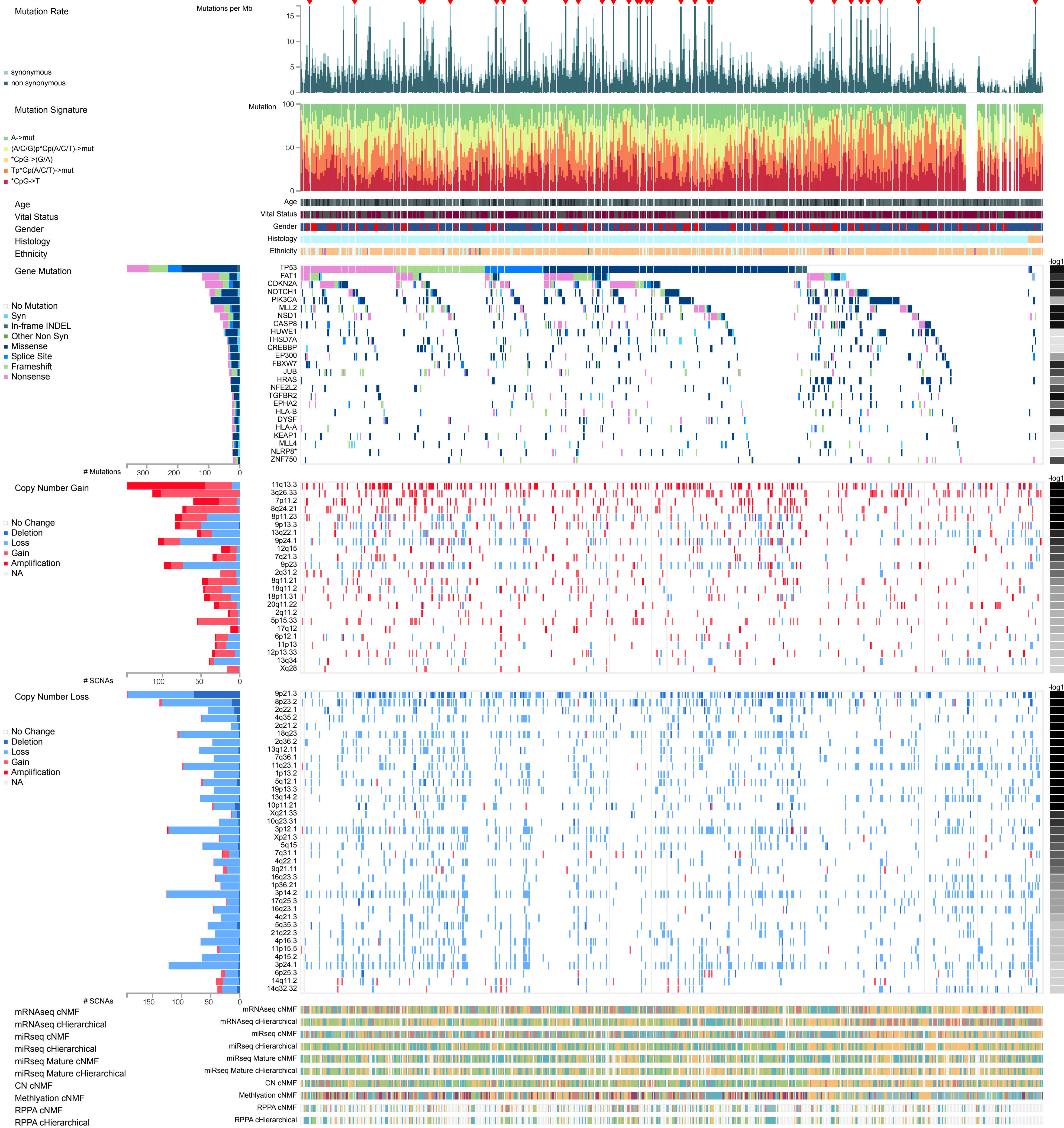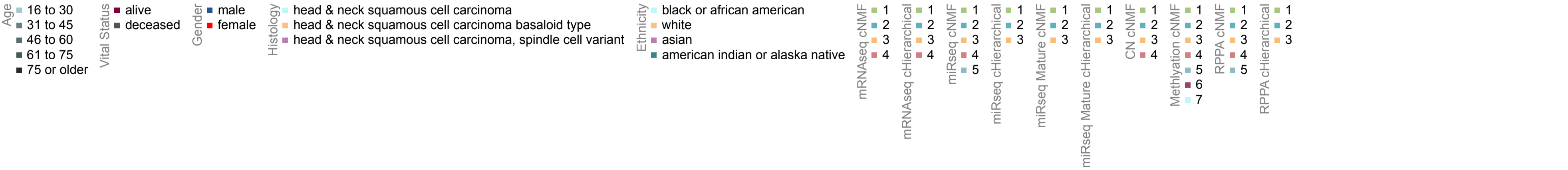

Supplement: Supplementary file 1 [file DataSheet_1.zip › Supplementary materials/Fig.S3/HNSC.pdf]

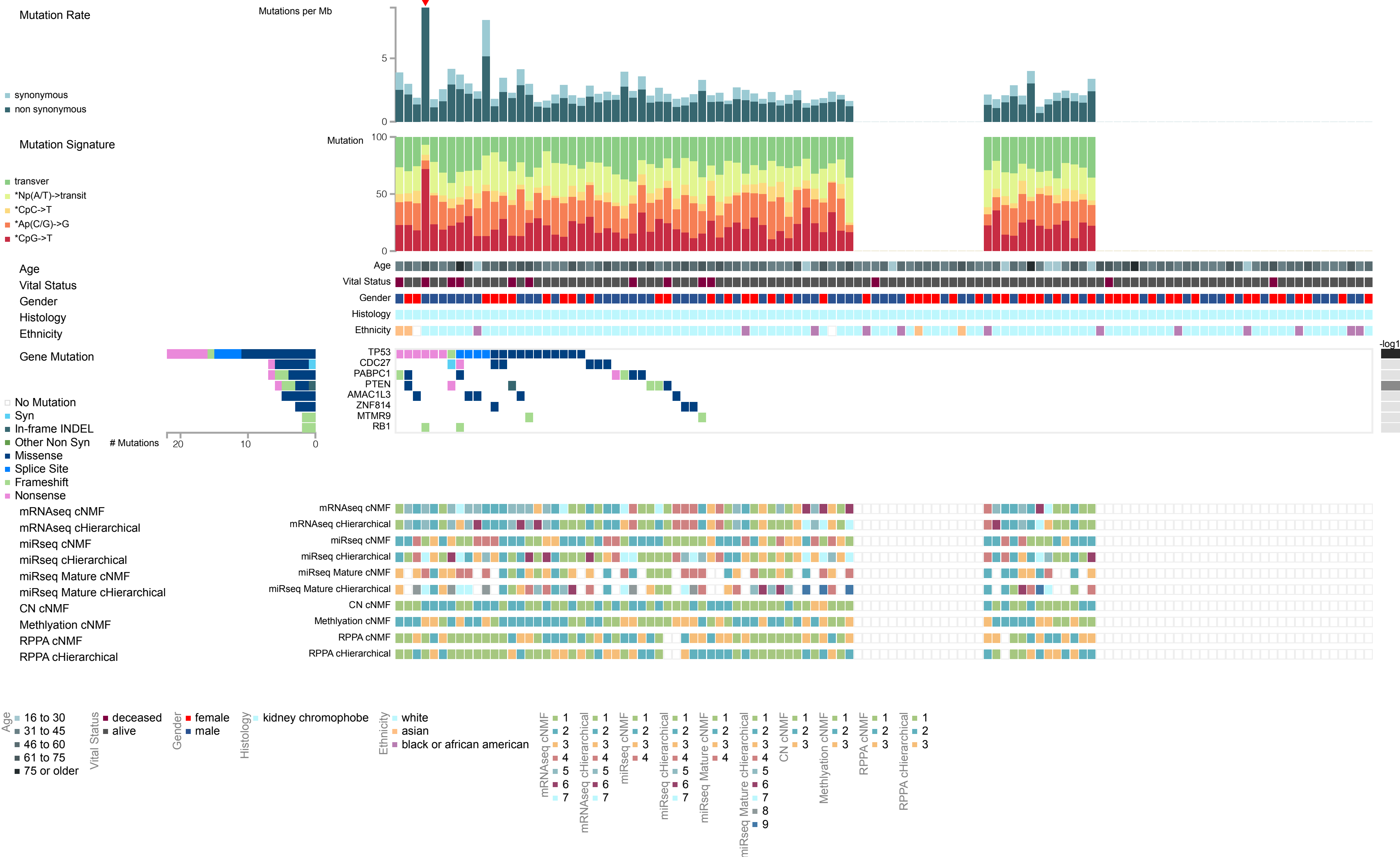

Supplement: Supplementary file 1 [file DataSheet_1.zip › Supplementary materials/Fig.S3/KICH.pdf]

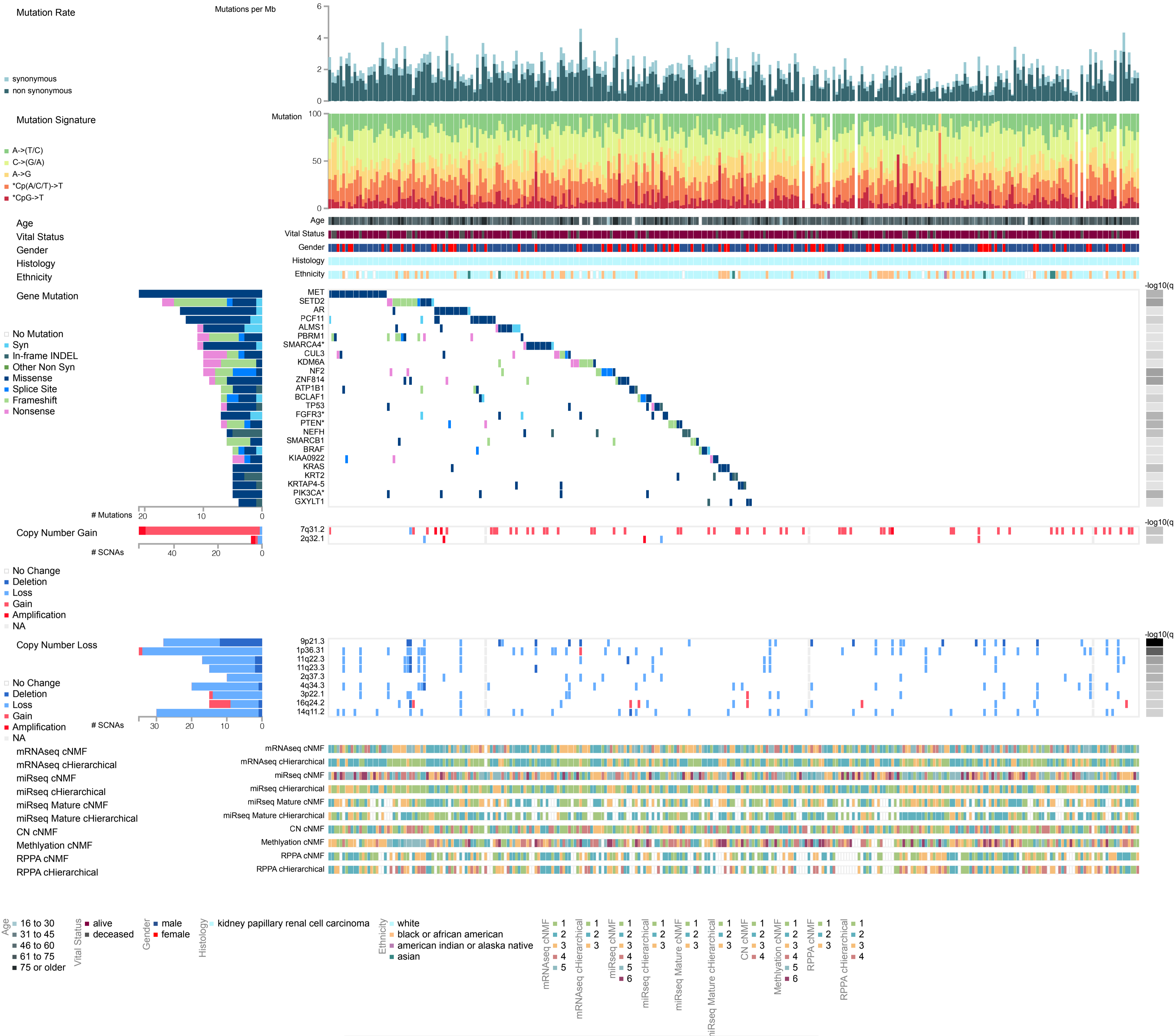

Supplement: Supplementary file 1 [file DataSheet_1.zip › Supplementary materials/Fig.S3/KIRP.pdf]

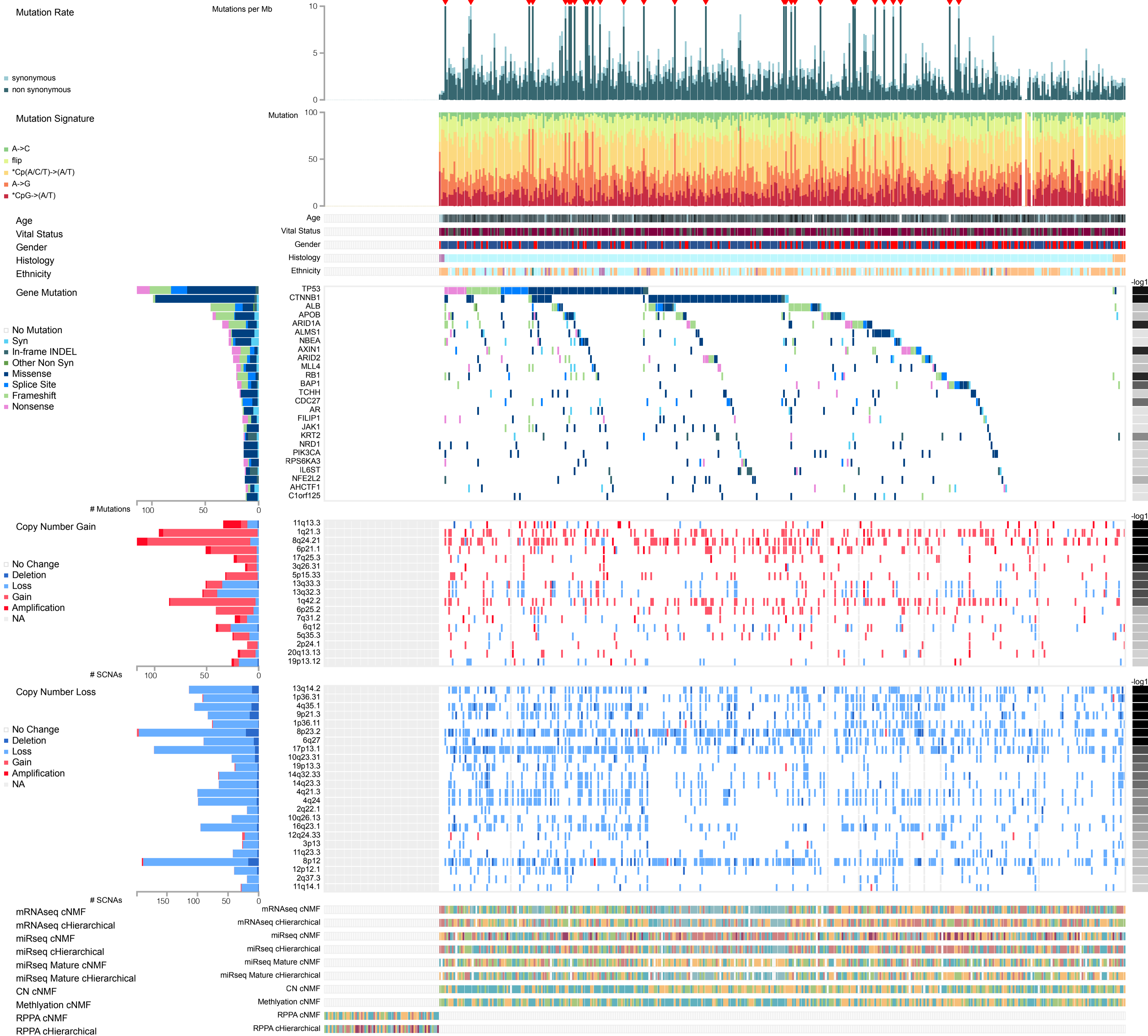

Supplement: Supplementary file 1 [file DataSheet_1.zip › Supplementary materials/Fig.S3/LIHC.pdf]

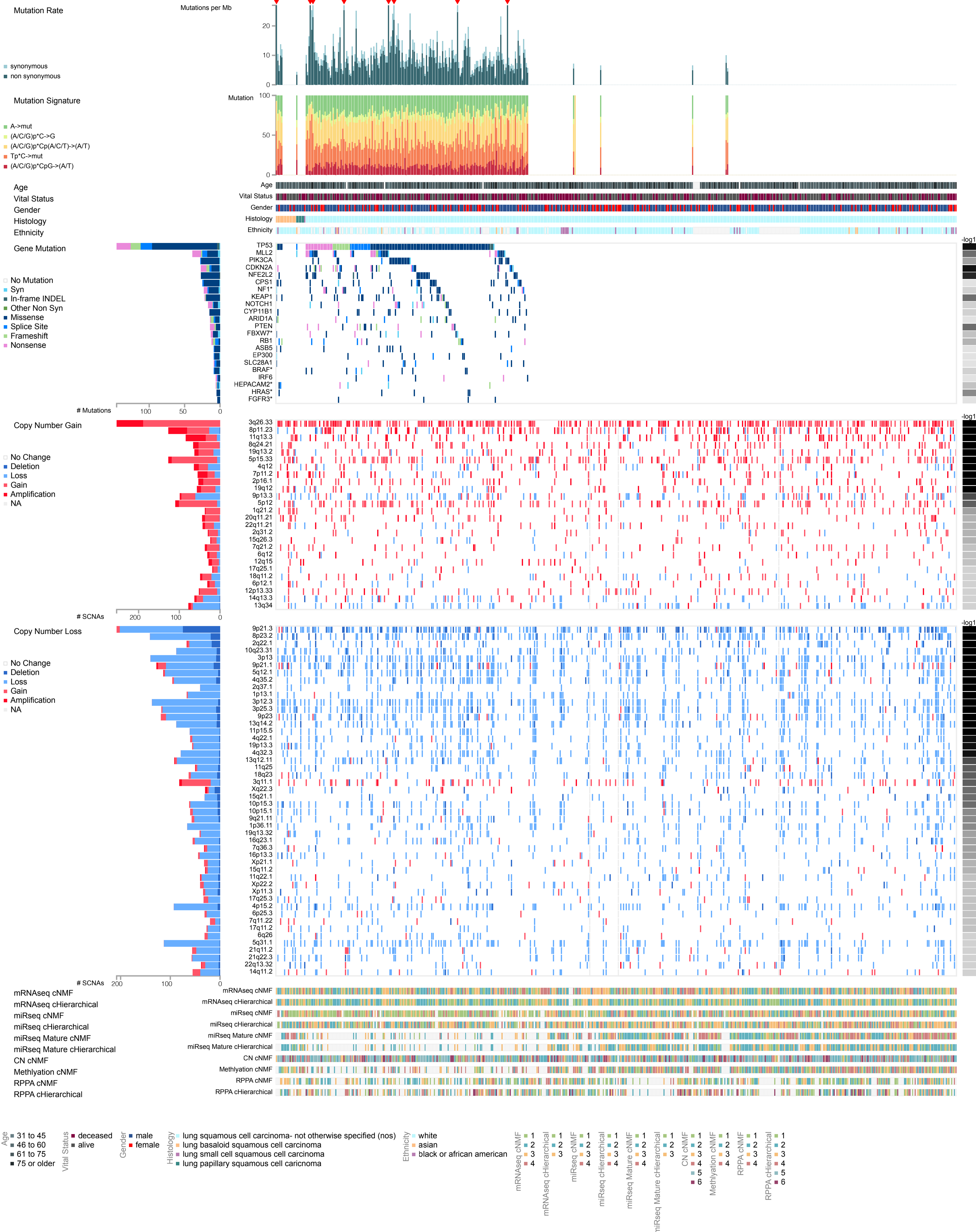

Supplement: Supplementary file 1 [file DataSheet_1.zip › Supplementary materials/Fig.S3/LUSC.pdf]

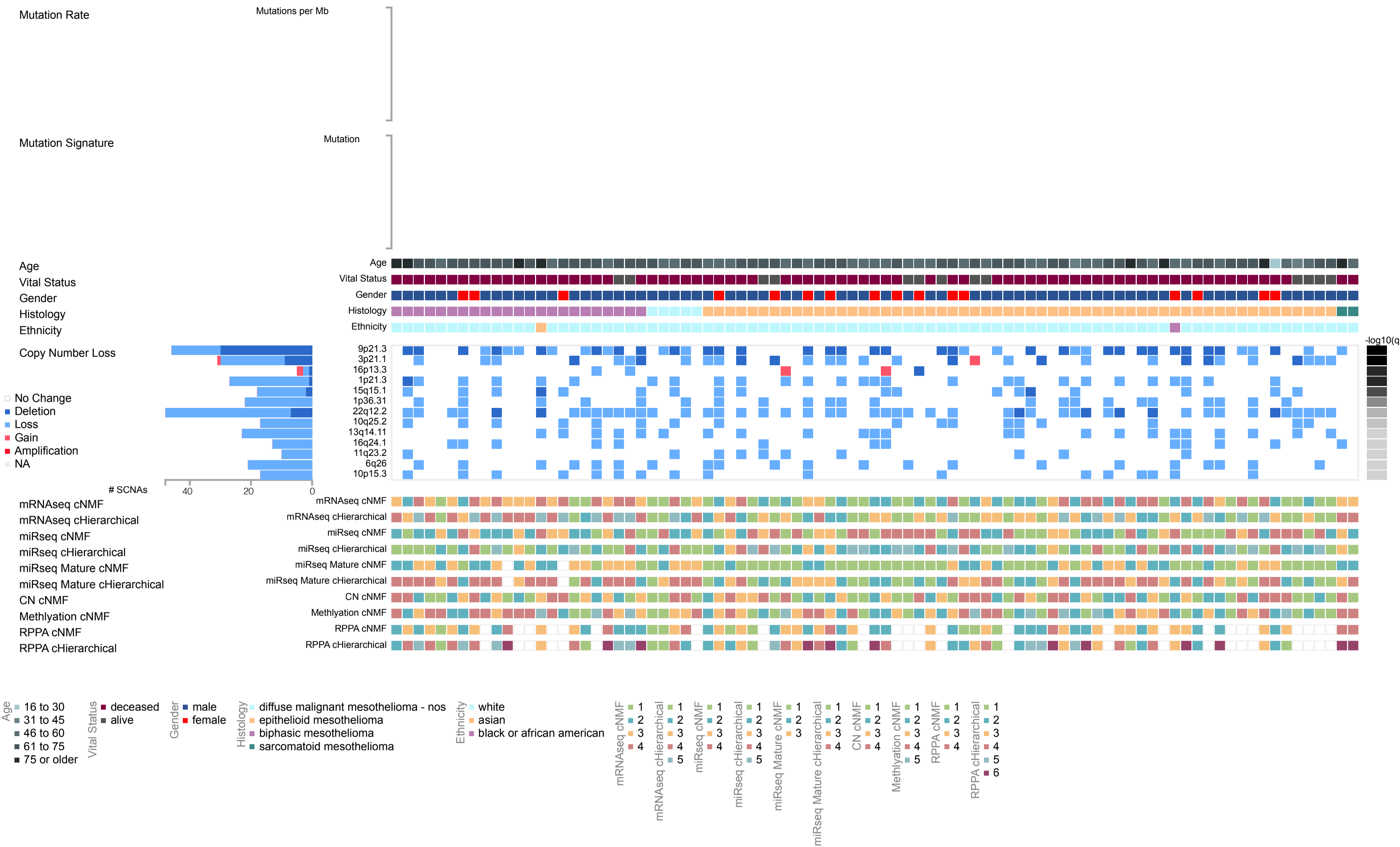

Supplement: Supplementary file 1 [file DataSheet_1.zip › Supplementary materials/Fig.S3/MESO.pdf]

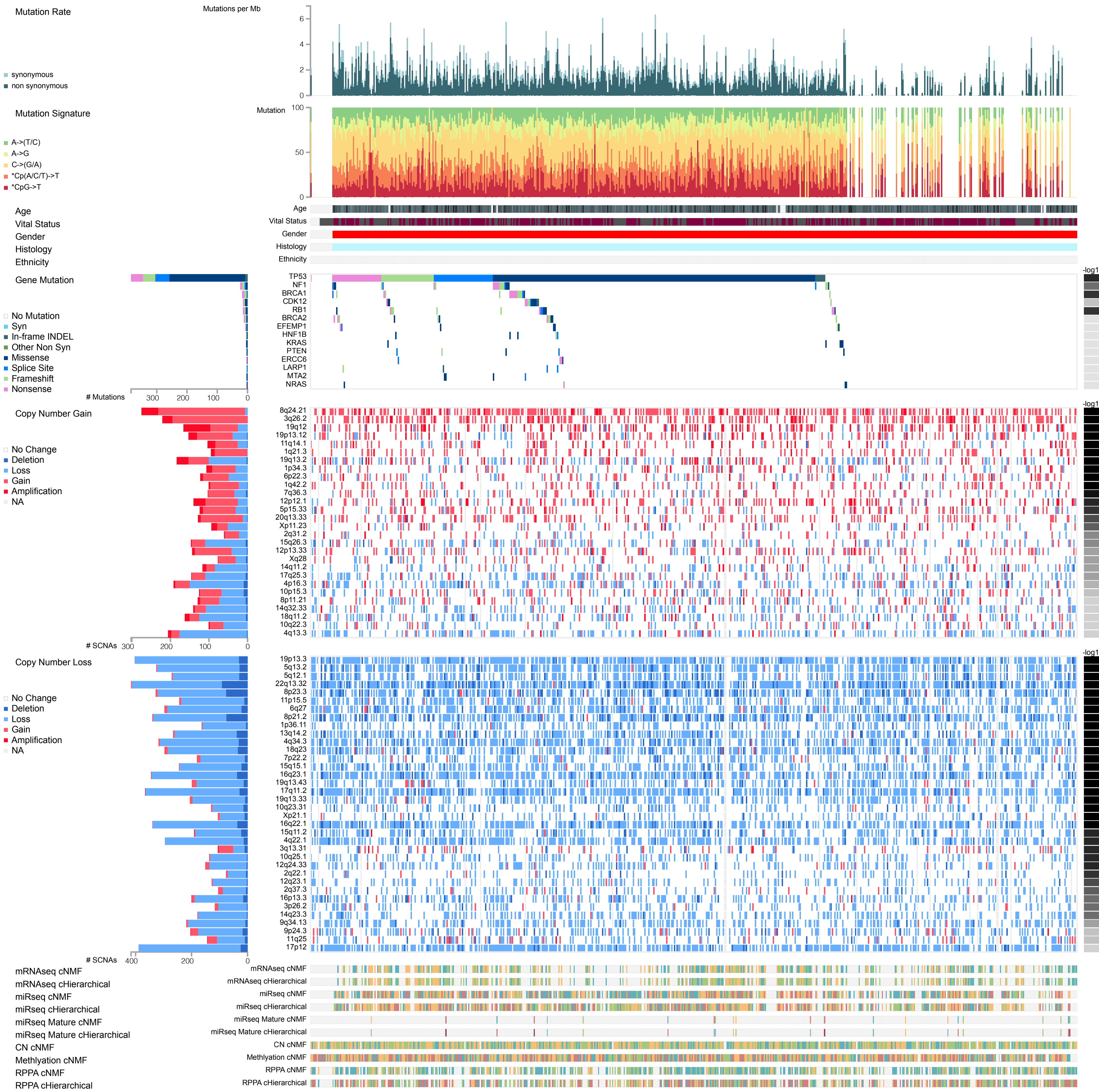

Supplement: Supplementary file 1 [file DataSheet_1.zip › Supplementary materials/Fig.S3/OV.pdf]

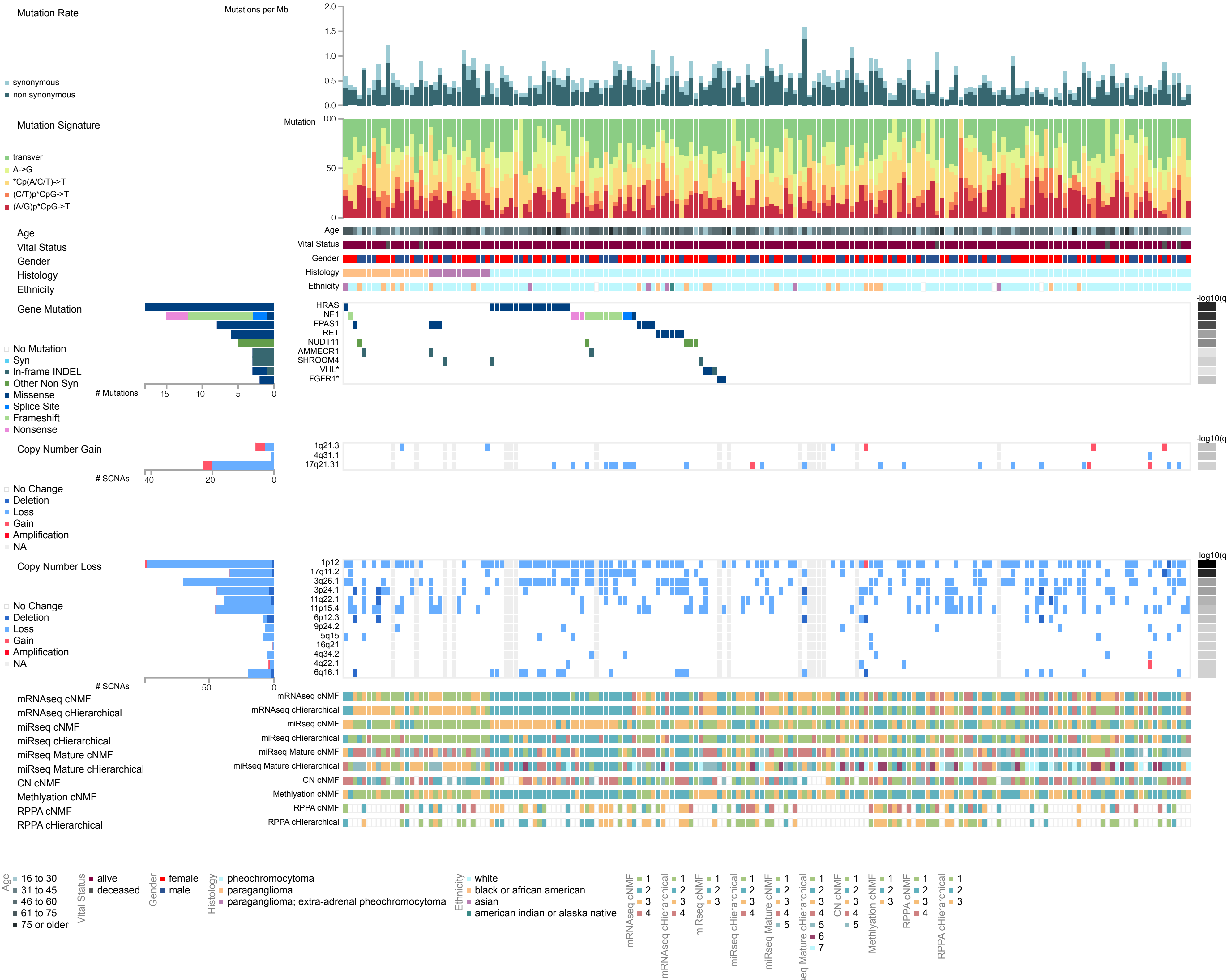

Supplement: Supplementary file 1 [file DataSheet_1.zip › Supplementary materials/Fig.S3/PCPG.pdf]

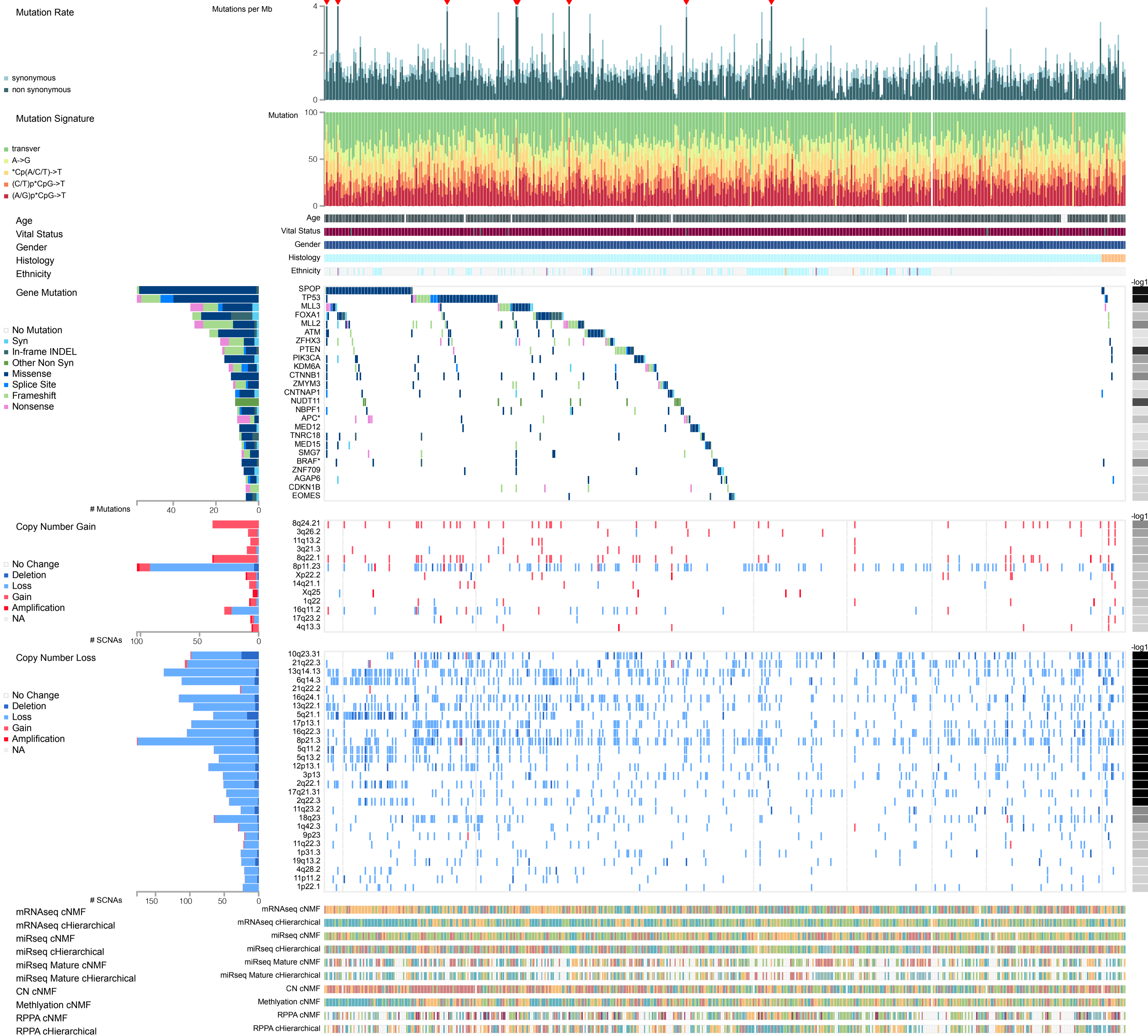

Supplement: Supplementary file 1 [file DataSheet_1.zip › Supplementary materials/Fig.S3/PRAD.pdf]

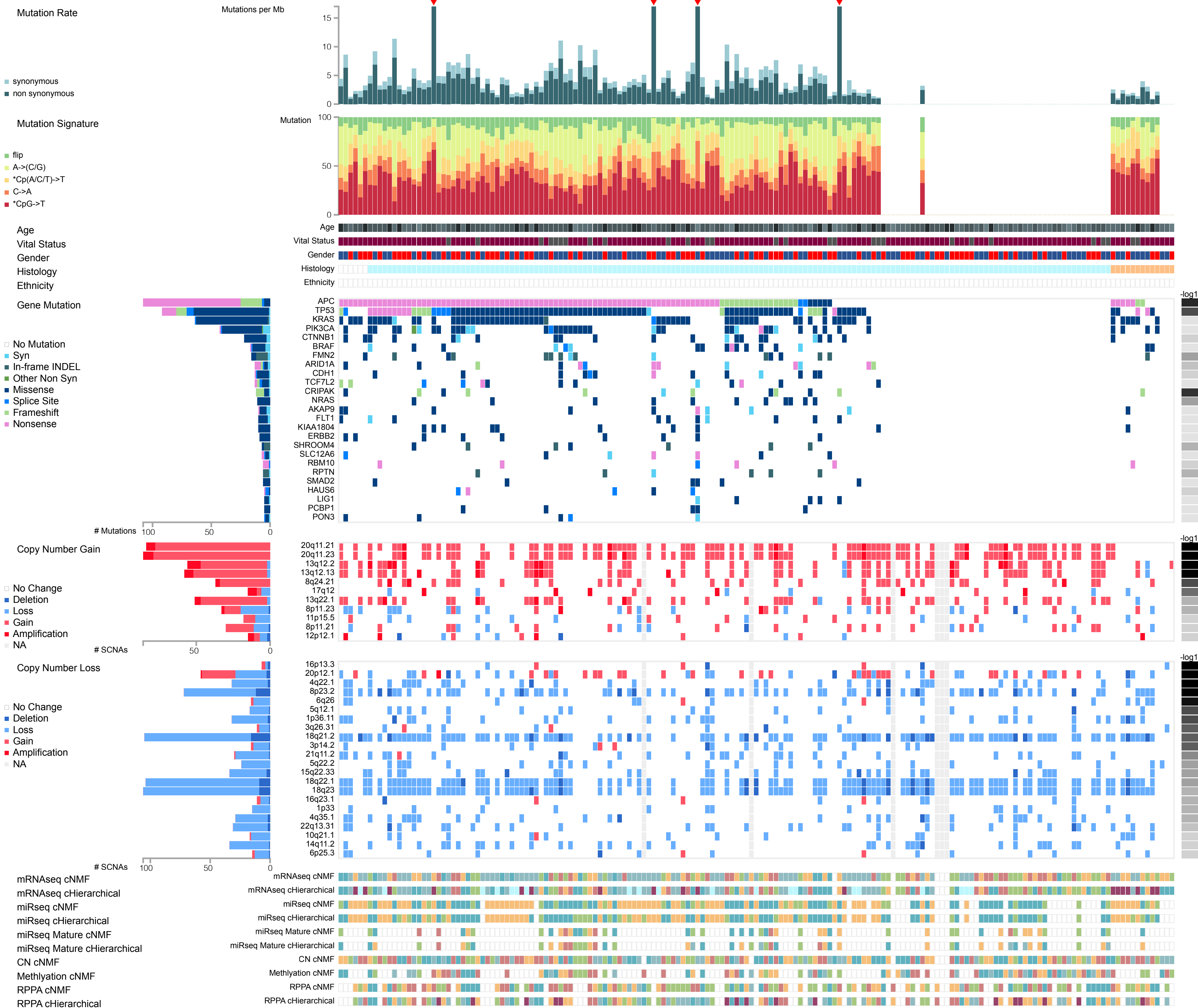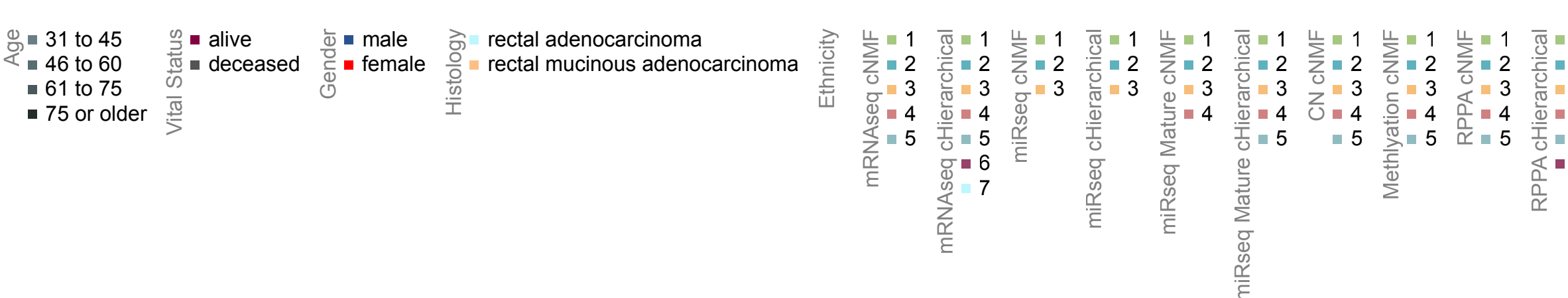

Supplement: Supplementary file 1 [file DataSheet_1.zip › Supplementary materials/Fig.S3/READ.pdf]

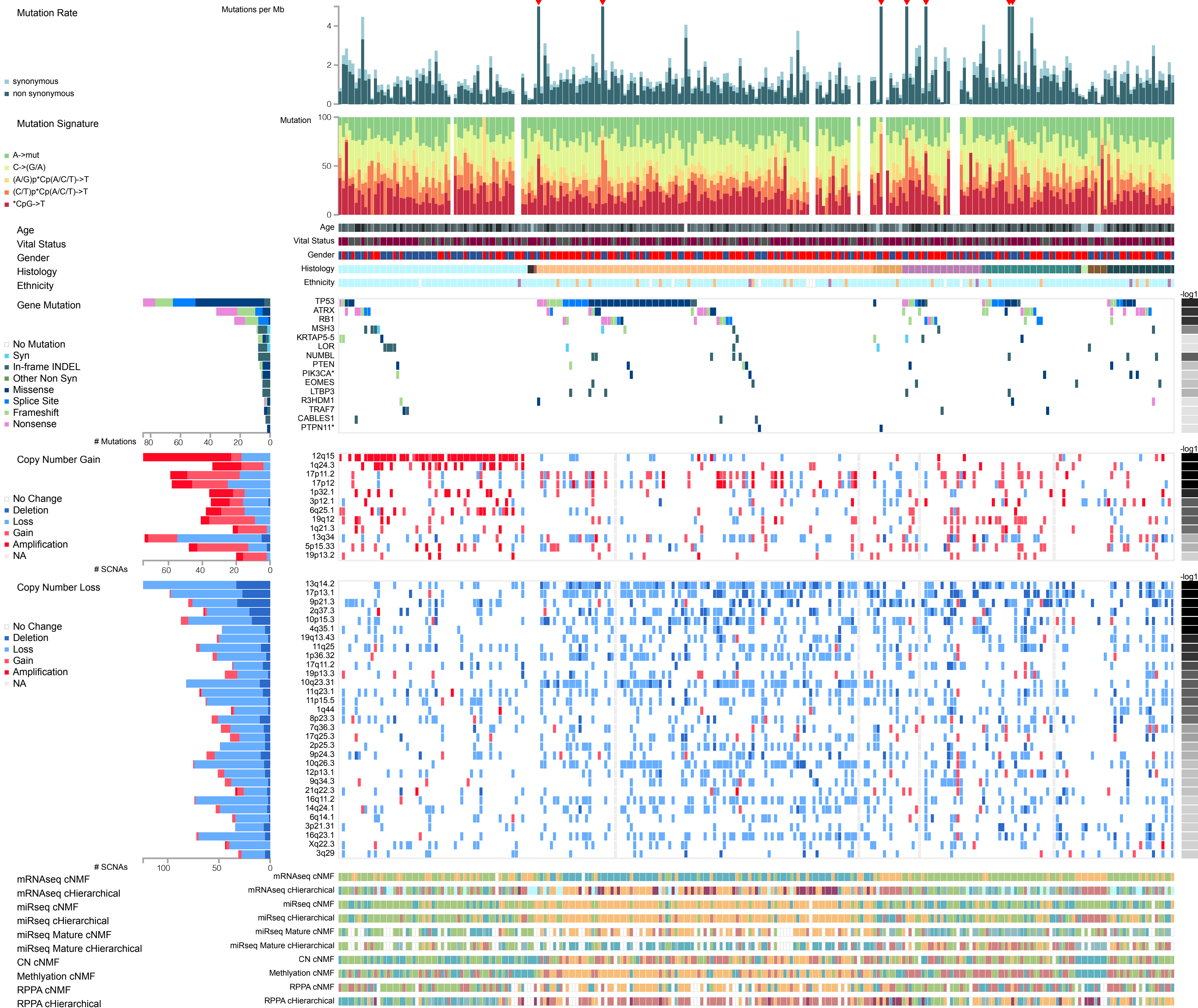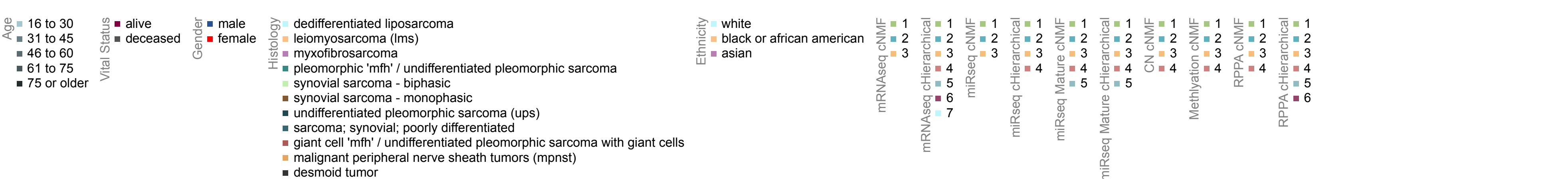

Supplement: Supplementary file 1 [file DataSheet_1.zip › Supplementary materials/Fig.S3/SARC.pdf]

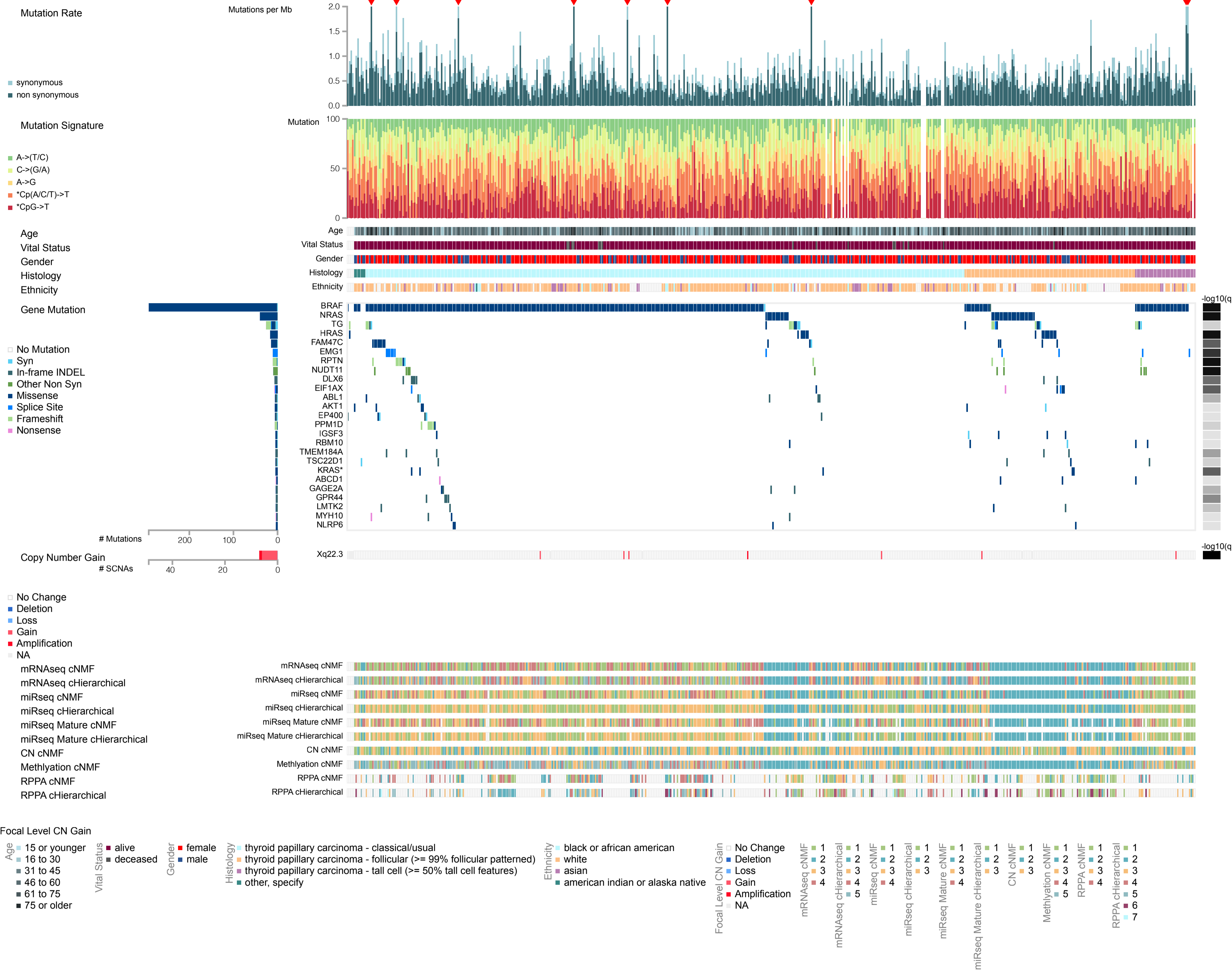

Supplement: Supplementary file 1 [file DataSheet_1.zip › Supplementary materials/Fig.S3/THCA.pdf]

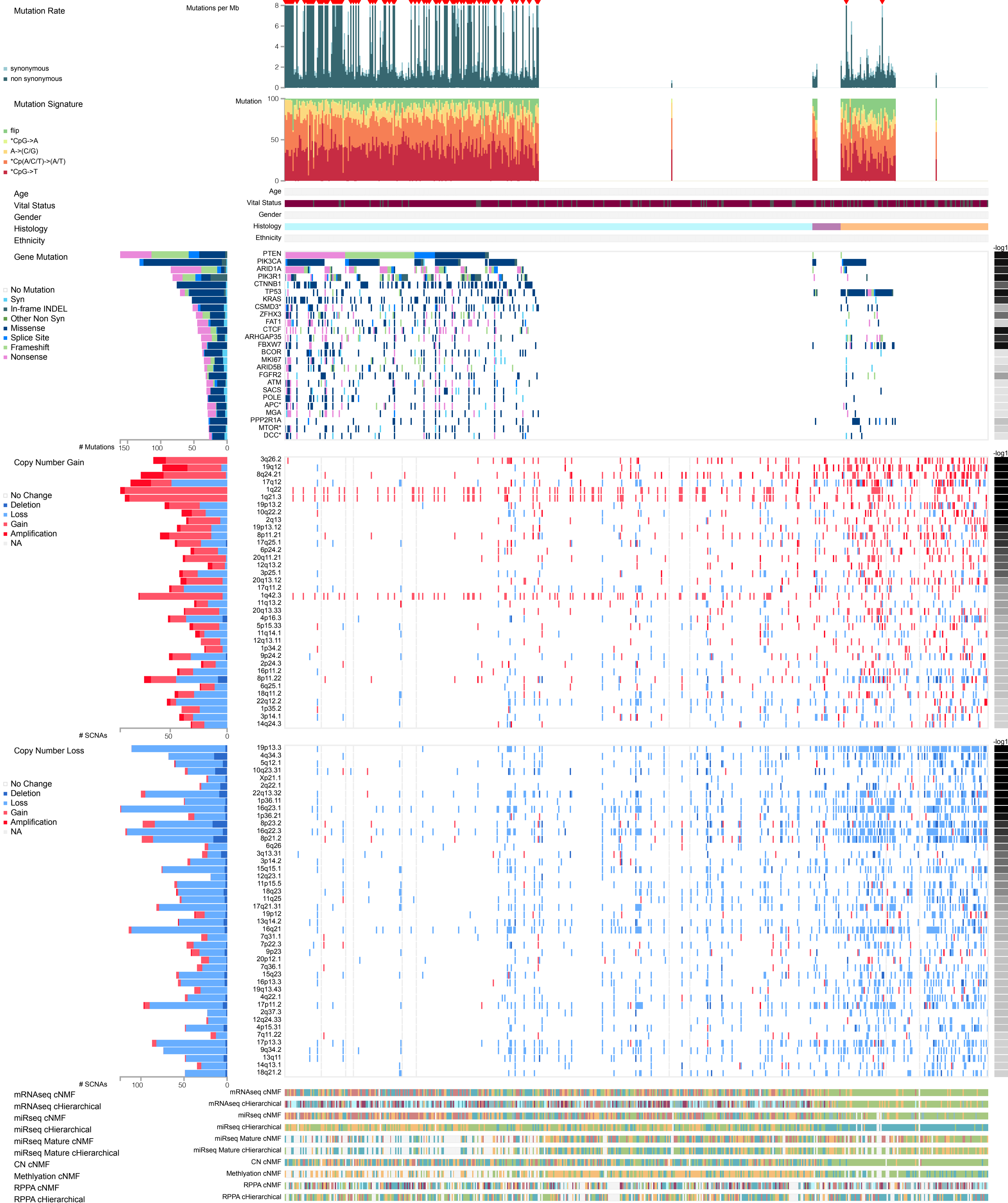

Supplement: Supplementary file 1 [file DataSheet_1.zip › Supplementary materials/Fig.S3/UCEC.pdf]

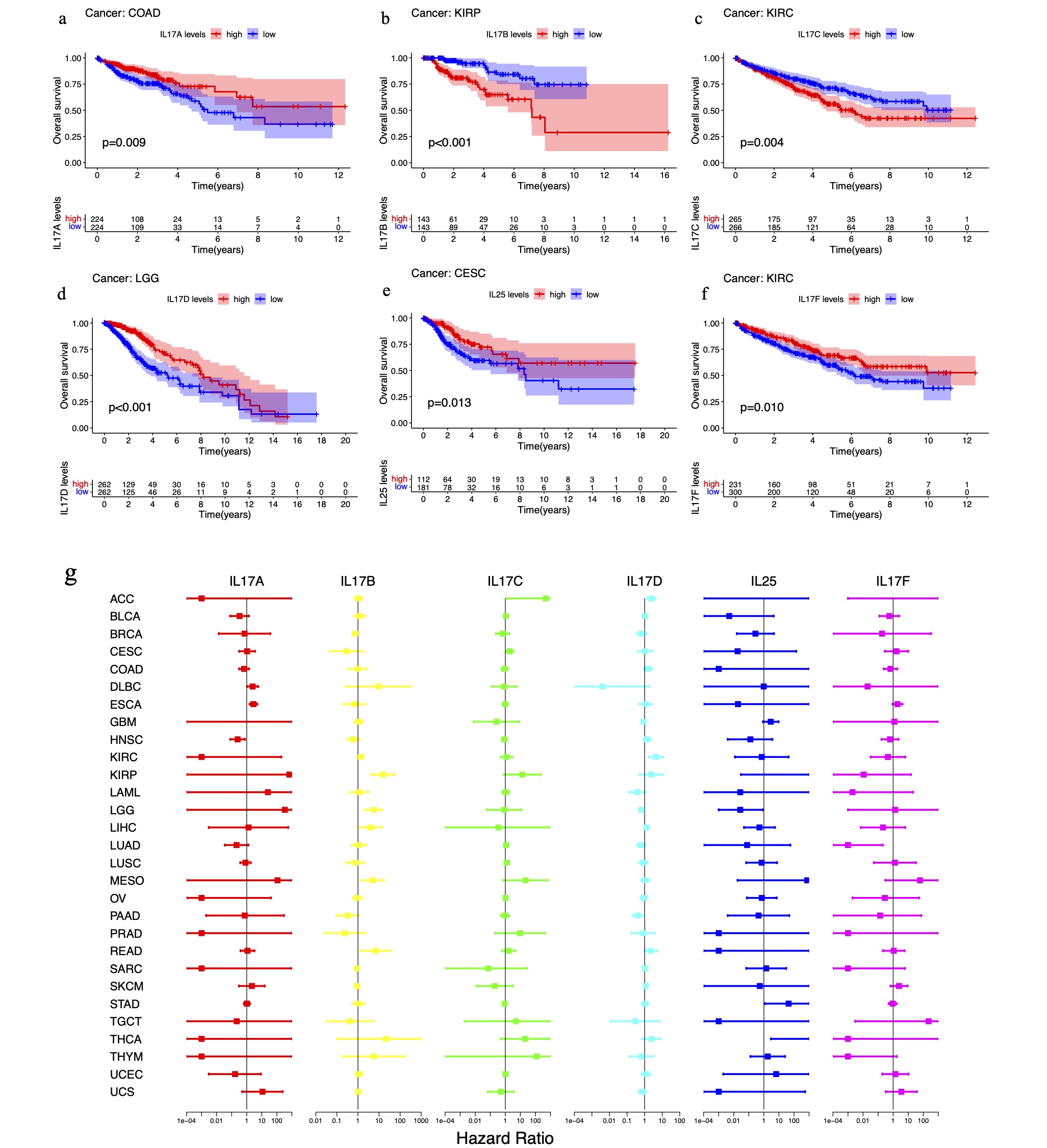

Supplement: Supplementary file 1 [file DataSheet_1.zip › Supplementary materials/Fig.S4.jpg]

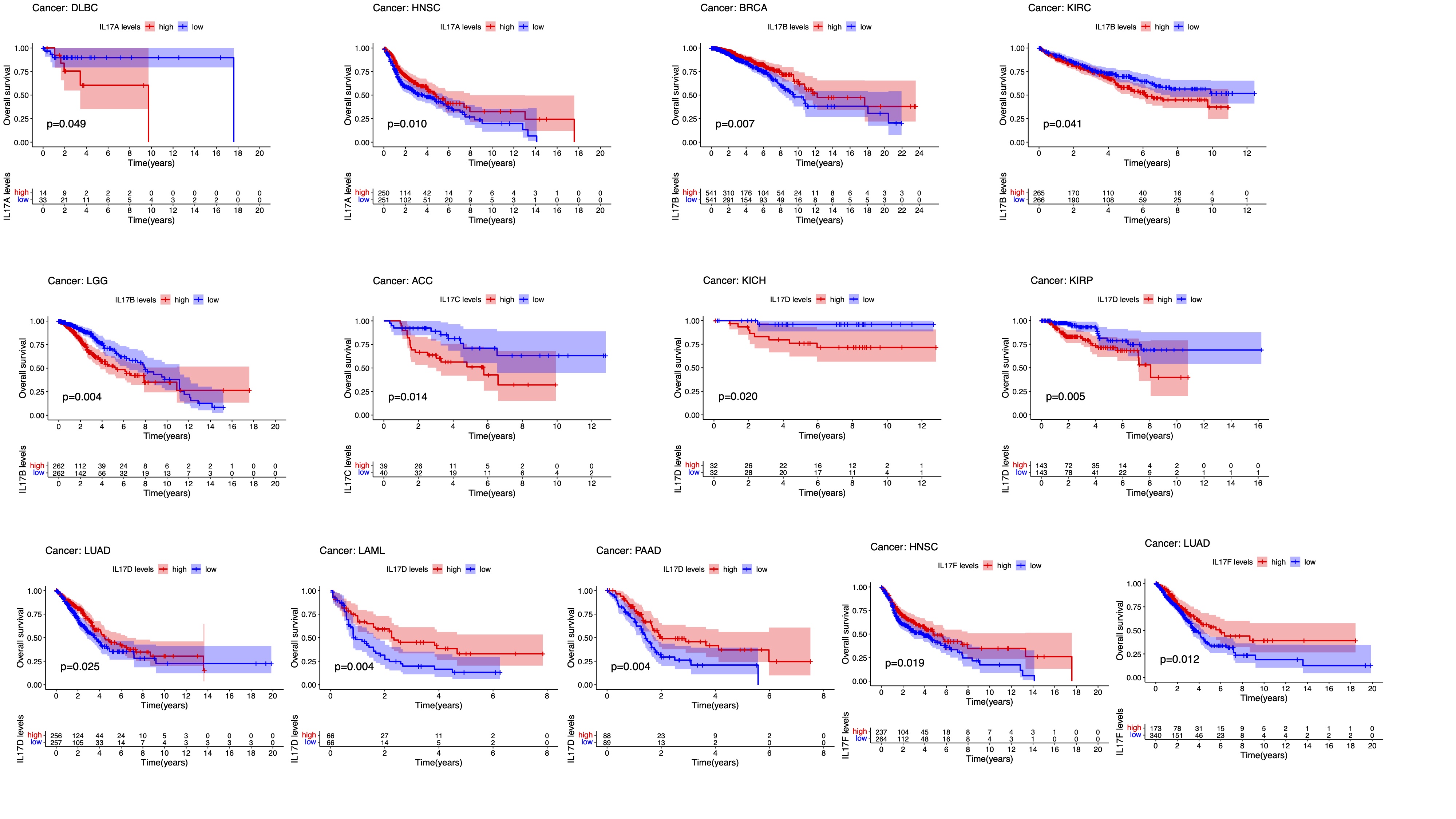

Supplement: Supplementary file 1 [file DataSheet_1.zip › Supplementary materials/Fig.S5 .jpg]

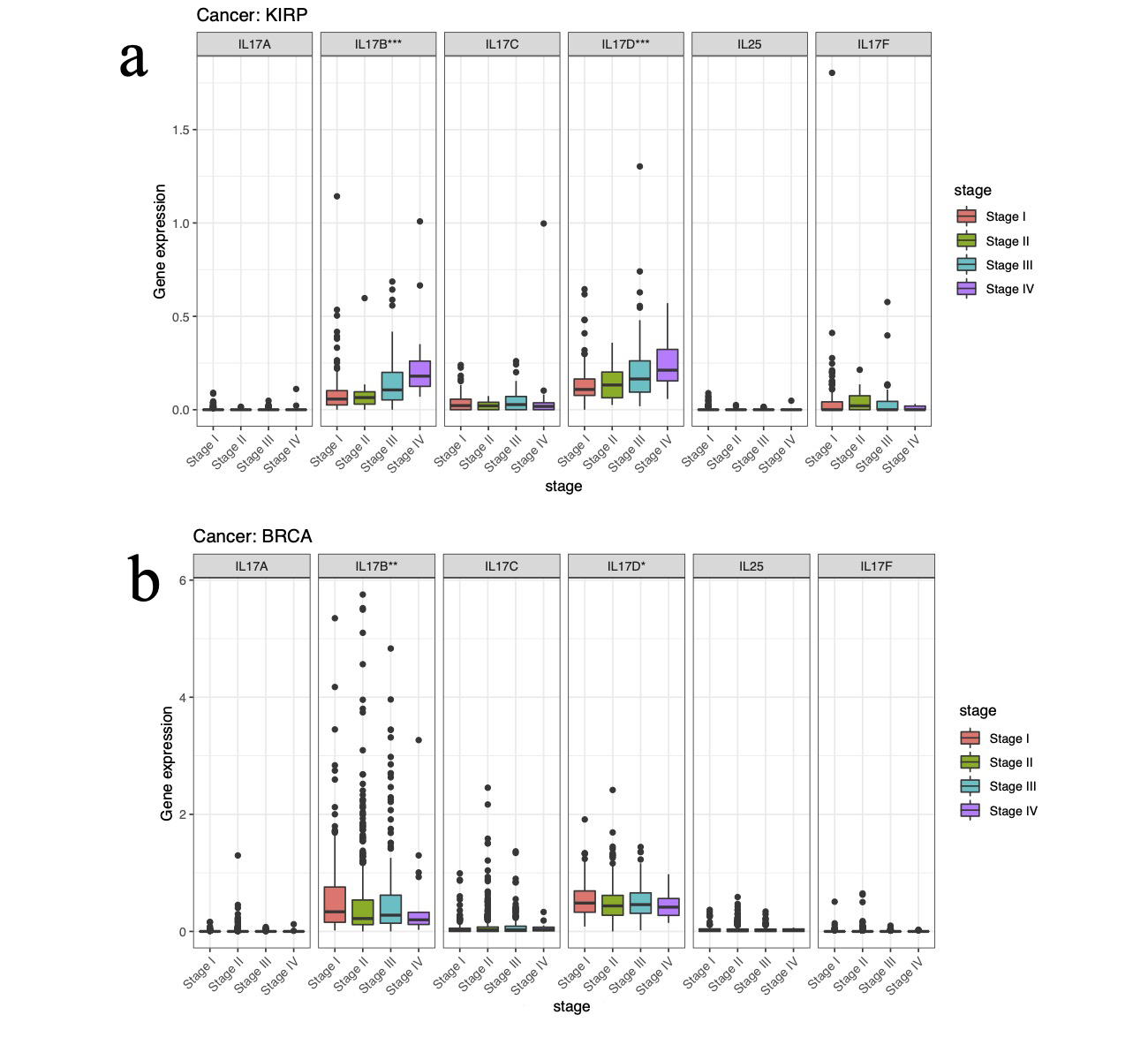

Supplement: Supplementary file 1 [file DataSheet_1.zip › Supplementary materials/Fig.S6.jpg]

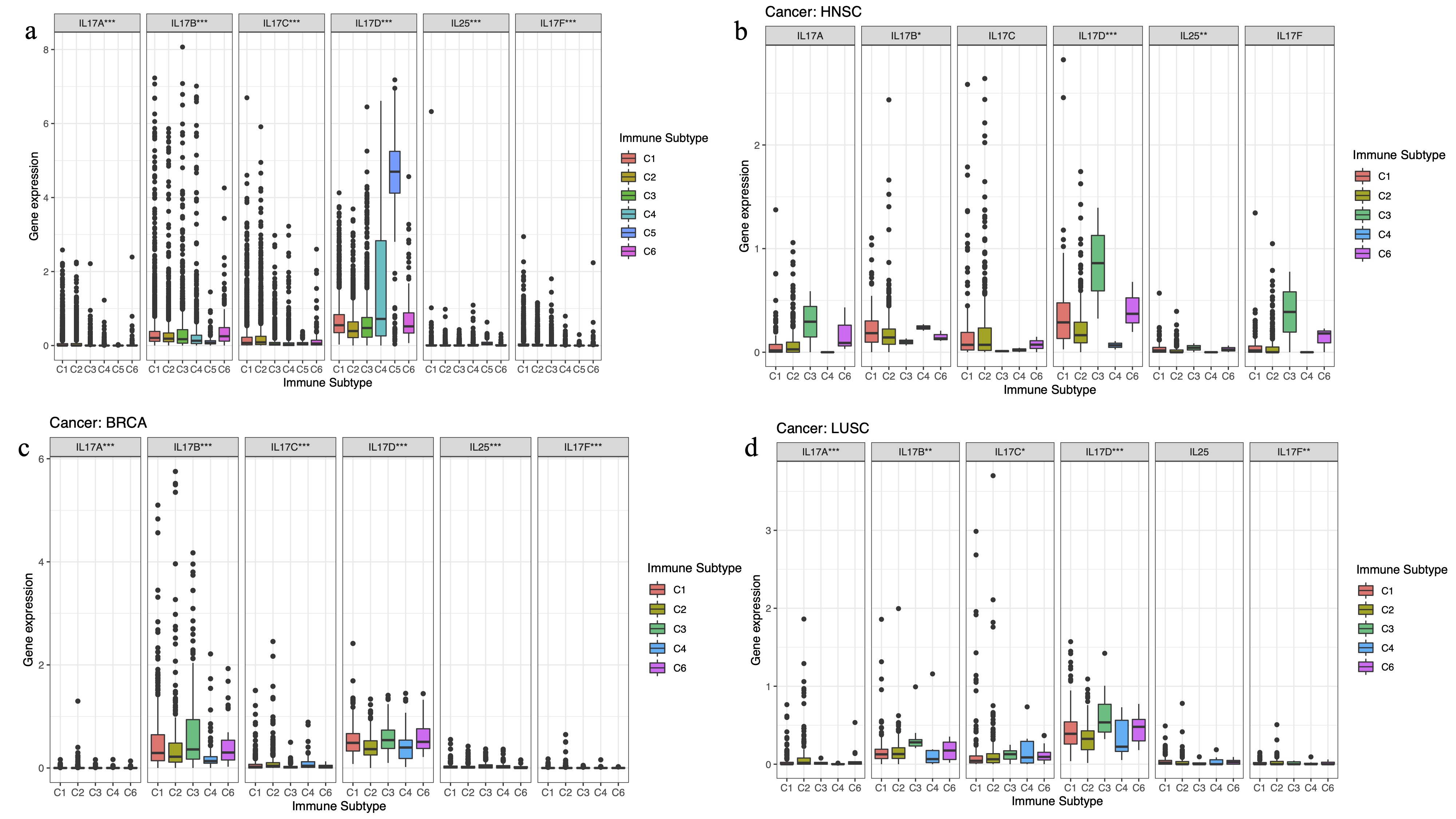

Supplement: Supplementary file 1 [file DataSheet_1.zip › Supplementary materials/Fig.S7.jpg]

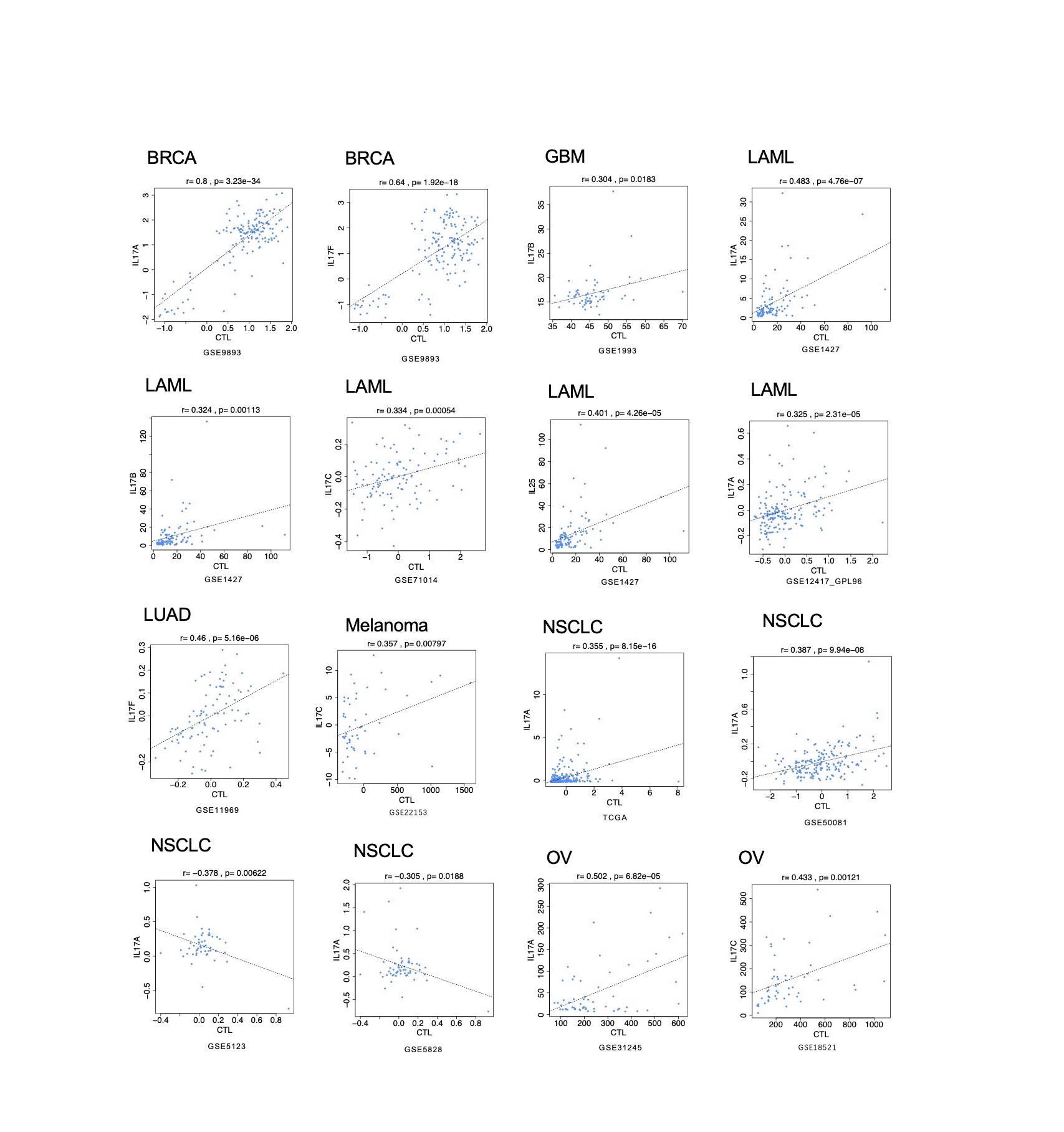

Supplement: Supplementary file 1 [file DataSheet_1.zip › Supplementary materials/Fig.S8.jpg]

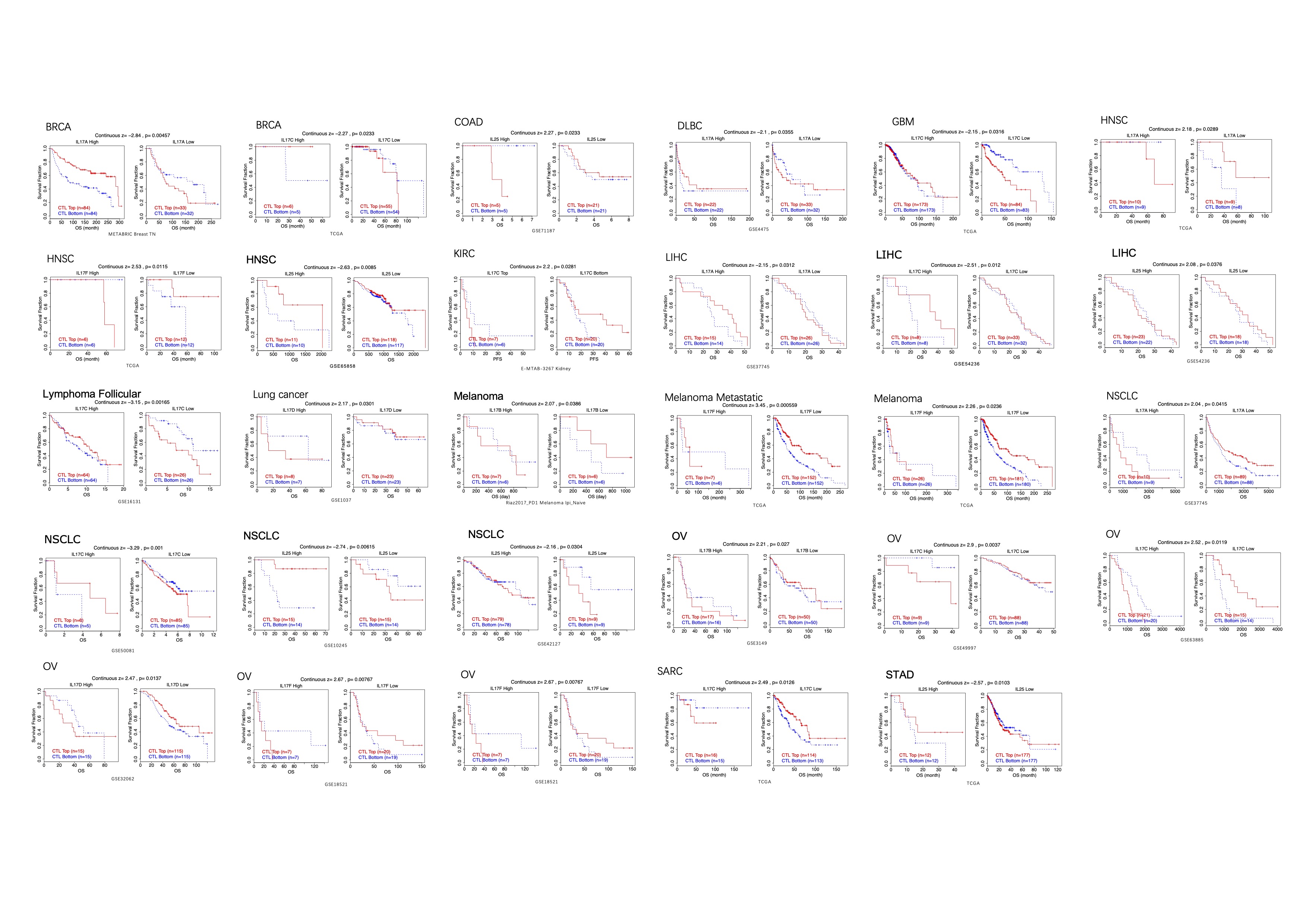

Supplement: Supplementary file 1 [file DataSheet_1.zip › Supplementary materials/Fig.S9 .jpg]
